# Taurine Is a Major Carbon and Energy Source for Marine Prokaryotes in the North Atlantic Ocean off the Iberian Peninsula

**DOI:** 10.1007/s00248-019-01320-y

**Published:** 2019-01-21

**Authors:** Elisabeth L. Clifford, Marta M. Varela, Daniele De Corte, Antonio Bode, Victor Ortiz, Gerhard J. Herndl, Eva Sintes

**Affiliations:** 1 Department of Limnology and Bio-Oceanography, Center of Functional Ecology, University of Vienna, Althanstrasse 14, 1090 Vienna, Austria; 2 Instituto Español de Oceanografía (IEO), Centro Oceanográfico de A Coruña, Apdo 130, 15080 A Coruña, Spain; 3 Research and Development Center for Marine Biosciences, Japan Agency for Marine-Earth Science and Technology (JAMSTEC), Natushima 2-15, Yokosuka, Kanagawa 237-0061 Japan; 4 Royal Netherlands Institute for Sea Research (NIOZ), Department of Marine Microbiology and Biogeochemistry, Utrecht University, PO Box 59, 1790 AB Den Burg, The Netherlands; 5 Instituto Español de Oceanografía (IEO), Centro Oceanográfico de Baleares, Moll de Ponent s/n, 07015 Palma de Mallorca, Spain

**Keywords:** Dissolved free taurine, Open ocean, Turnover rates, North Atlantic, Prokaryotic metabolism

## Abstract

Taurine, an amino acid-like compound, acts as an osmostress protectant in many marine metazoans and algae and is released via various processes into the oceanic dissolved organic matter pool. Taurine transporters are widespread among members of the marine prokaryotic community, tentatively indicating that taurine might be an important substrate for prokaryotes in the ocean. In this study, we determined prokaryotic taurine assimilation and respiration throughout the water column along two transects in the North Atlantic off the Iberian Peninsula. Taurine assimilation efficiency decreased from the epipelagic waters from 55 ± 14% to 27 ± 20% in the bathypelagic layers (means of both transects). Members of the ubiquitous alphaproteobacterial SAR11 clade accounted for a large fraction of cells taking up taurine, especially in surface waters. Archaea (Thaumarchaeota + Euryarchaeota) were also able to take up taurine in the upper water column, but to a lower extent than Bacteria. The contribution of taurine assimilation to the heterotrophic prokaryotic carbon biomass production ranged from 21% in the epipelagic layer to 16% in the bathypelagic layer. Hence, we conclude that dissolved free taurine is a significant carbon and energy source for prokaryotes throughout the oceanic water column being utilized with similar efficiencies as dissolved free amino acids.

**Electronic supplementary material:**

The online version of this article (10.1007/s00248-019-01320-y) contains supplementary material, which is available to authorized users.

## Introduction

Prokaryotes are the main consumers of dissolved organic matter (DOM) in the ocean [1], and consequently play a central role in the ocean’s biogeochemical cycles [2]. Among the plethora of DOM compounds, dissolved free amino acid (DFAA) species are typically present in pico- and nanomolar concentrations in ocean waters and hence represent only a minor fraction of the oceanic DOM pool [3]. However, DFAA are rapidly recycled and can be significant sources of carbon (C), nitrogen (N), and energy for heterotrophic prokaryotes. Depending on the trophic state of the system and prokaryotic community composition, DFAA can contribute between ~ 6 and 51% to the N- [4–6] and between ~ 2 and 37% to the C demand of heterotrophic prokaryotes in the ocean [6–8].

Taurine (2-aminoethanesulfonic acid), an amino acid-like compound, belongs to the naturally occurring organosulfonates [9]. Together with glycine and alanine, taurine is common in marine metazoans and in their release products [10, 11]. For example, taurine can constitute up to ~ 74% of the free amino acid pool in clam tissue [12, 13] and up to 40% of the free amino acid pool in the release products of mesozooplankton [10, 11]. Taurine is also produced by a large number of algae [14, 15]. Besides diverse (cyto-) protective roles (e.g., antioxidant, detergent, signaling molecule), taurine and its derivatives and conjugates are well known for their key role in counteracting hydrostatic pressure in marine organisms [16, 17].

Prokaryotes are the main consumers of taurine [9, 18]. The ability to utilize taurine has been reported for specific bacterial representatives of diverse taxa [18, 19]. Dissolved taurine can potentially be a source of C, N, sulfur (S), and energy [18], as reported for prokaryotes in microbial mats and sediments [19–21]. Additionally, taurine degradation can also be a significant source of other metabolically important S and N species (sulfate, sulfide, bisulfide, thiosulfate, ammonium, alanine, and other organosulfonates) [18, 22, 23]. In general, oganosulfonates can account for 20–40% of the total organic S in marine sediments [24]. However, our knowledge on the role of taurine as a substrate and energy source for open ocean prokaryotic communities is rather limited.

In the oceanic water column, taurine is present in similar concentrations as DFAA species, i.e., in the low nanomolar range in surface and coastal waters, and in the picomolar range or below the detection limit in bathypelagic waters [11, 25]. Taurine transporters and enzymes involved in the taurine metabolism are widespread in natural prokaryotic communities of surface [26] and deep waters [27, 28]. Genes encoding for taurine transporters and enzymes involved in taurine metabolism in the world’s oceans have been mainly affiliated to the abundant alphaproteobacterial taxa Rhodobacteraceae and the SAR11 clade [29] contributing up to 30% and 50% to the prokaryotic community, respectively [30, 31]. Simon et al. [30] suggest that taurine is used as C and energy source by Rhodobacteraceae rather than as a S-source in the surface ocean. However, taurine is also an important S-source for some heterotrophic bacterioplankton species such as for the abundant SAR11 clade [32, 33]. In the epipelagic layer, particularly in the deep chlorophyll maximum, cyanobacteria might also be important consumers of taurine [34, 35]. Also members of other relevant shallow and deep-water taxa appear to have the potential to utilize taurine [35, 36]. Remarkably, there is no information available on the uptake rates and turnover of taurine in natural marine prokaryotic communities in the open ocean. Our poor understanding on the turnover of taurine in marine pelagic systems is in striking contrast with the well-studied turnover of DFAAs and their importance as a substrate for marine prokaryotes [7, 37].

In this study, we measured dissolved taurine concentrations and prokaryotic taurine respiration and assimilation rates throughout the water column (0–5000 m) of two transects off the Galician coast in the North Atlantic Ocean (northwestern Spain). The comparison between total heterotrophic prokaryotic biomass production, assessed via leucine incorporation, and taurine assimilation allowed us to determine the relative importance of taurine as a substrate for planktonic prokaryotes. Our results indicate that epi- and upper mesopelagic bacterioplankton assimilate taurine more efficiently than bathypelagic prokaryotes and that taurine represents a significant source of C and energy for the prokaryotic community throughout the oceanic water column.

## Material and Methods

### Study Area and Sampling

**Fig. 1:**
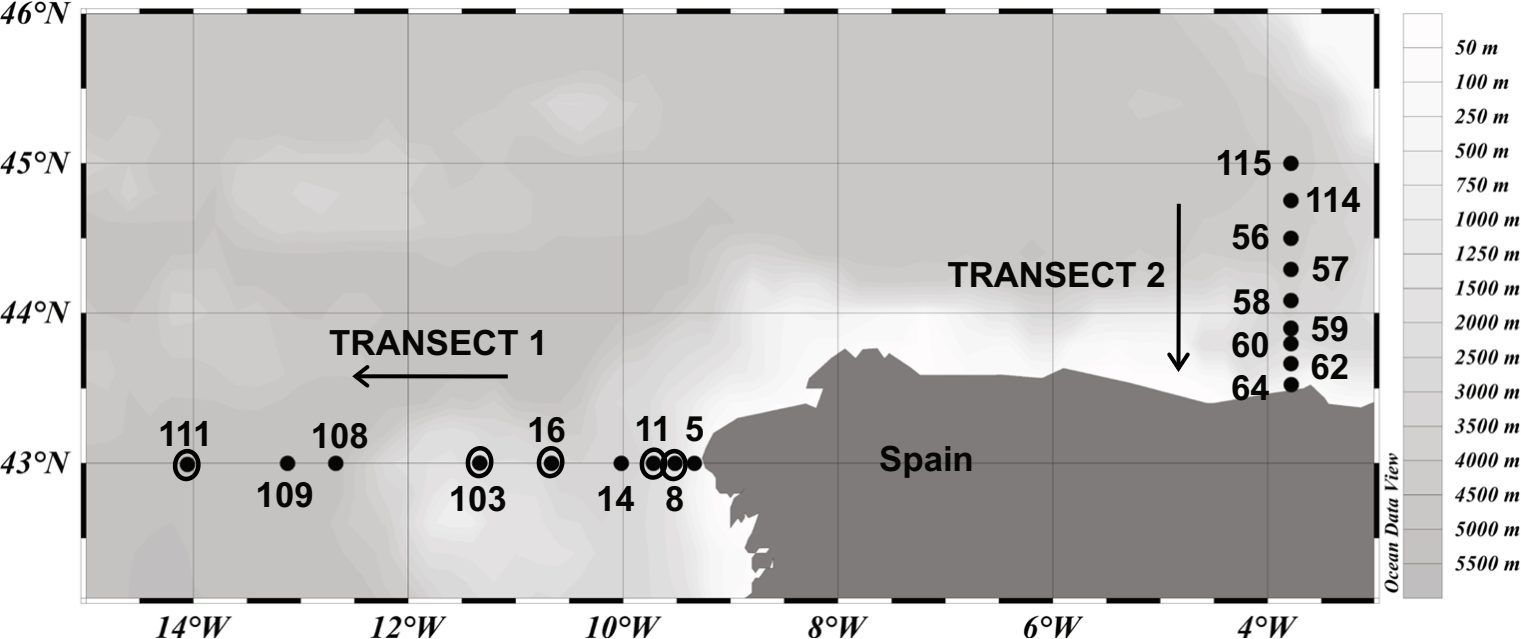
Sampling stations in the North Atlantic. Dots indicate the sampling stations occupied during the MODUPLAN cruise and black open circles indicate sampling stations occupied during the RadProf cruise. The MODUPLAN cruise took place in August 2014 and the RadProf cruise in August 2015. During the MODUPLAN cruise, sampling was conducted at transect 1 from 08/4/2014 to 08/12/2014 and at transect 2 from 08/14/2014 to 08/21/2014. Arrows indicate direction of sampling. During RadProf cruise, sampling was conducted from 08/01/2015 to 08/07/2015

Sampling was conducted in the North Atlantic during the MODUPLAN cruise with R/V *Sarmiento de Gamboa* in August 2014 and during the RadProf cruise with R/V *Ramon Margalef* in August 2015. Water samples were collected with a CTD (conductivity, temperature, depth) rosette sampler holding 12 L Niskin bottles at 18 stations (MODUPLAN) along two different transects (one off the Galician coast and one in the Bay of Biscay; northwestern Iberian Peninsula; Fig. 1). Five of the stations of transect 1 were revisited during the RadProf cruise. Seawater was sampled from surface to bathypelagic layers at 6 to 10 depths per station. Water was transferred from the Niskin bottles into 100–500 mL polycarbonate flasks for DFAA and taurine analyses, prokaryotic abundance and biomass production, bulk taurine assimilation and respiration measurements, and microautoradiography in combination with catalyzed reporter deposition fluorescence in situ hybridization (MICRO-CARD-FISH). All the samples were immediately processed as described below.

### Determining Prokaryotic Abundance

Water samples (1.5 mL) were fixed with glutaraldehyde (0.5% final concentration) at room temperature for 10 min. After flash freezing in liquid nitrogen for 10 min, water samples were stored at − 80 °C. Before analysis, water samples were thawed to room temperature. Subsamples (0.5 mL) were taken and stained with SYBR Green I (1× final concentration) in the dark for 10 min. As an internal standard, 1 μm fluorescent beads (Molecular Probes, 1 × 10^5^ mL^−1^ final conc.) were added to the subsamples. Prokaryotic cells were counted on a BD FacsAria II flow cytometer based on their signature in a plot of green fluorescence versus side scatter [38, 39].

### Determining Dissolved Organic Carbon, Free Taurine, and Dissolved Free Amino Acid Concentrations

Samples to determine dissolved organic carbon (DOC) concentrations were collected at each depth and processed as previously described [11] using a Shimadzu TOC-V_CSH_ analyzer [40]. To determine dissolved free taurine and dissolved free amino acid (DFAA), 5 mL water samples were collected. Water samples were taken from the 100-mL polycarbonate flasks with 20-mL syringes and gently filtered through pre-rinsed 0.2 μm pore-size Acrodisc filters (25 mm; Pall, Supor membrane) into pre-combusted (at 450 °C for 4 h) glass vials and subsequently stored at − 20 °C until further analysis. Analysis were performed using high-pressure liquid chromatography (HPLC) and fluorescence detection after pre-column ortho-phthalaldehyde derivatization as described in Clifford et al. [11]. Concentrations of taurine and 19 DFAA species were measured. The LOD (limit of detection), LOQ (limit of quantification), the linearity (*R*^2^), and the recovery (%) for taurine and the DFAA species measured are given in Table S1.

### Prokaryotic Taurine Assimilation and Respiration Measurements

To determine taurine assimilation and respiration rates, duplicate water samples (40 mL) and one formaldehyde-killed control were spiked with ^14^C-taurine ([1,2-^14^C], Biotrend, specific activity 60 mCi/mmol; final concentration 10 nM) and incubated in ~ 120 mL biological oxygen demand (BOD) flasks in the dark at in situ temperature for 5–54 h, depending on the expected activity [41]. The 10 nM taurine concentration added to the samples represents the saturating substrate concentration (Fig. S1a).

The BOD flasks were sealed with a rubber stopper holding a plastic cup in the headspace of the flasks containing a filter paper wick (Whatman no. 1). Incubations were terminated by adding 0.8 mL sulfuric acid (2 N H_2_SO_4_) injected with a syringe through the rubber stopper to acidify the sample. Subsequently, 0.2 mL of phenethylamine was added to the paper wick by injection through the rubber stopper to trap the ^14^CO_2_ originating from the respired ^14^C-taurine. To facilitate the trapping of the ^14^CO_2_, BOD flasks were placed on a laboratory shaker at room temperature and agitated for 1–2 h. The acidified water samples were filtered onto 0.2 μm polycarbonate filters (Millipore GTTP, 25 mm diameter) and rinsed twice with 10 mL Milli-Q water. Subsequently, the filters and the paper wicks were placed in scintillation vials, air-dried, and 8 mL of Filter Count scintillation cocktail (Perkin Elmer) added to each vial. After 18 h, the disintegrations per minute (DPM) were determined in a scintillation counter (TriCarb 2800 TR Liquid Scintillation Analyzer, Perkin Elmer).

The obtained DPM were used to determine taurine assimilation and respiration rates. Taurine assimilation was calculated from the DPM collected on the filter, taurine respiration from the DPM obtained from the paper wick, and taurine uptake rates represent the sum of taurine assimilation and respiration. The DPM of the two replicates from each sample were averaged and DPM from the corresponding killed control were subtracted. Taurine assimilation and respiration rates were corrected for the external isotope dilution using the taurine concentration measured in the water samples from the corresponding depth. Taurine turnover rates were calculated by dividing taurine uptake rates (assimilation plus respiration) by the in situ concentrations of dissolved taurine determined by HPLC. The taurine assimilation efficiency of a given sample was calculated by dividing the taurine assimilation rate by the taurine uptake rate. Cell-specific uptake and assimilation rates were calculated by dividing the measured uptake and assimilation rate, respectively, by the in situ prokaryotic cell abundance of a given sample.

### Leucine Incorporation Measurements

During the MODUPLAN cruise, ^3^H-leucine incorporation into prokaryotic protein was measured using two different methods. The centrifugation method was used on samples above 1000 m depth [42] for its convenience [43]. For samples below 1000 m with typically low prokaryotic activity, the filtration method was used as described elsewhere [42]. Both methods used ^3^H-leucine (3,4,5-^3^H L-leucine, Biotrend, specific activity 120 Ci/mmol) at a final concentration of 5 nM, which represents saturation substrate concentration (Fig. S1b). During the RadProf cruise, only the filtration method was used, and the incubation time and volume were adjusted depending on the expected prokaryotic abundance and activity. Briefly, duplicate water samples (5–40 mL) and one formaldehyde-killed control were inoculated with ^3^H-leucine and incubated in the dark at in situ temperature for 6–24 h. Subsequently, the samples were fixed with formaldehyde (2% final concentration), filtered onto 0.2 μm polycarbonate filters (Millipore GTTP, 25 mm diameter), and rinsed twice with 10 mL of ice-cold 5% trichloroacetic acid (TCA). Then, the filters were air-dried and transferred into scintillation vials. Eight milliliters of Filter Count scintillation cocktail (Perkin Elmer) was added to each vial and the DPM determined in a Tricarb 2800 TR Liquid Scintillation Counter (Perkin Elmer). The DPM of the blank were subtracted from the mean DPM of the respective sample. The resulting DPM were converted into leucine incorporation rates taking the external isotope dilution factor into account. The dissolved free leucine concentration measured in the corresponding water samples by HPLC was used to determine the external isotope dilution factor for each sample. Leucine turnover rates were determined by dividing leucine incorporation rates by the in situ concentrations of dissolved free leucine. Cell-specific leucine incorporation rates were calculated by dividing the measured incorporation rate by the prokaryotic cell abundance of the specific sampling station and depth.

### Microautoradiography in Combination with Catalyzed Reporter Deposition Fluorescence In Situ Hybridization

In order to determine the possible variations in taurine uptake of different prokaryotic taxa under contrasting environmental conditions, samples were collected at two contrasting stations of transect 1 during the MODUPLAN cruise. Station 8 was located at the continental slope and thus in the upwelling area and station 111 was off the continental slope in the open Atlantic (Fig. 1). Microautoradiography in combination with catalyzed reporter deposition fluorescence in situ hybridization (MICRO-CARD-FISH) was used to identify and determine the abundance of specific prokaryotic taxa taking up taurine. Water samples (10–40 mL) and one formaldehyde-killed control (2% final concentration) were spiked with ^3^H-taurine (specific activity 40 Ci/mmol, Biotrend) at a final concentration of 10 nmol L^−1^ and incubated in the dark at in situ temperature for 5–54 h, depending on the expected activity based on previous measurements of prokaryotic abundance and activity at the sampling site. Incubations were terminated by adding 0.2 μm filtered formaldehyde (2% final concentration). Subsequently, the fixed samples were stored at 4 °C in the dark for 12–18 h. Thereafter, the samples were filtered onto a 0.2-μm polycarbonate filter (Millipore, GTTP) supported by a nitrocellulose filter (Millipore, HAWP, 0.45 μm), washed twice with Milli-Q water, air-dried, and stored in a microfuge vial at − 20 °C until further processing in the home laboratory. The method used for processing the filters is described elsewhere [44, 45]. The oligonucleotide probes, the hybridization conditions, and the target phylogenetic groups used for this approach are given in Table S2. Multiple probes were combined to hybridize SAR11, Thaumarchaeota, and Bacteria as previously reported [46–48] to cover the broad diversity of prokaryotic taxa. Following CARD-FISH hybridization, filters were exposed to photographic emulsion (Carestream NTB) at 4 °C. An experiment was also conducted to evaluate the effect of exposure time on the relative abundance of cells surrounded by a silver grain halo using water samples from two different depths. Filters from these two samples were exposed for 1, 2, 3, 4, 5, 7, 10, and 12 days. The number of cells with associated silver grains did not further increase after 10 days of exposure. Consequently, we routinely used an exposure time of 12 days for all the analyses. The slides were developed and fixed according to the manufacturer’s instructions, and cells counterstained with a DAPI-mix as previously described [44]. The slides were examined with an Axio Imager M2 (Carl Zeiss) microscope equipped with a 100-W Hg lamp and appropriate filter sets for DAPI and Alexa 488. The presence of silver grains surrounding the cells was determined using the transmission mode of the microscope. Cells with two or more associated silver grains were considered to actively take up taurine (Fig. S2). In formaldehyde-killed controls, less than 0.5% of the total DAPI-stained cells were associated with silver grains. For each microscopic field, we enumerated the DAPI-stained cells, the cells hybridized with the specific oligonucleotide probe, DAPI-stained cells with associated silver grains, and probe-specific hybridized cells with associated silver grains. Cell-specific taurine uptake rates were calculated by dividing the bulk taurine uptake rate by the abundance of prokaryotic (DAPI-stained) cells taking up taurine at the corresponding depth.

### Contribution of Taurine-C, -N, and -S to Prokaryotic Biomass Production

To estimate the contribution of taurine assimilation to the heterotrophic prokaryotic biomass production, we converted the measured taurine assimilation rates to taurine-derived C assimilated. Leucine incorporation rates were converted to heterotrophic prokaryotic C-biomass production by applying the theoretical conversion factor of 1.55 kg C mol^−1^ (assuming no isotope dilution) [49]. Additionally, assuming that the new biomass production calculated from the leucine results in atomic ratios of C:N of 4:1 and C:S of 26:1 [50] of newly formed cells, we calculated the potential contribution of taurine-N and taurine-S to the N- and S-requirements of heterotrophic marine prokaryotes.

### Global Distribution of Taurine Uptake Genes

To assess the global distribution of potential taurine uptake by prokaryotes, metagenomics data from the Tara Oceans expedition from the fractions 0.2–1.6 μm or 0.2–3 μm were used (Table S3) [51]. Prokaryotic KEGG abundance (http://ocean-microbiome.embl.de/companion.html) from taurine transporters *tau*A (K15551), *tau*B (K10831), and *tau*C (K15552) was normalized by the sum of *rec*A (K03553) and *rad*A (K04483) to account for Bacteria and Archaea, respectively. Environmental data and data analyses are described in detail elsewhere [51, 52].

## Results

### Physico-chemical Characteristics of the Water Column

Potential temperature–salinity diagrams for the different stations are shown in Fig. S3. Surface seawater temperatures varied between 20 and 22 °C and decreased with depth down to approximately 2.5 °C. Lowest salinity was detected in surface waters in transect 1 (~ 34.5) and below 1200 m depth (~ 35.5), corresponding to Labrador Seawater and North Atlantic Deep Water [53]. The highest salinity (transect 1 ~ 36.1; transect 2 ~ 36.1) was measured at around 1000 m depth corresponding to Mediterranean Sea Outflow Water. The North Atlantic Central Water was located between 200 and 500 m with a salinity of ~ 35.6 and a temperature range of 11–12 °C. The oxygen minimum was located at ~ 1000 m depth (~ 180 μmol kg^−1^).

### Dissolved Free Amino Acids, Leucine, and Taurine Concentrations Throughout the Water Column

The sum of the concentration of DFAA (Fig. S4a, b and Table S4) was significantly higher for the epipelagic realm (mean values of both transects: 32.4 ± 19.2 nM, *n* = 73, *p* < 0.05, Kruskal–Wallis test followed by a Mann–Whitney *U* test) than for the mesopelagic (19.3 ± 16.9, *n* = 54) and bathypelagic layer (17.6 ± 9.4 nM, *n* = 49) (*p* < 0.05). Mean DFAA concentrations in transect 2 were lower at all depth layers (epipelagic: 26.4 ± 8.5 nM, *n* = 30; mesopelagic: 11.9 ± 5.3 nM, *n* = 21; bathypelagic: 11.4 ± 4.7 nM, *n* = 17) than in transect 1 (epipelagic: 36.6 ± 21.5 nM, *n* = 43; mesopelagic: 24.2 ± 20.0 nM, *n* = 32; bathypelagic: 20.6 ± 9.7 nM, *n* = 33). However, the sum of DFAA of transect 2 was significantly lower only in the meso- and the bathypelagic layers than in transect 1 (*p* < 0.05, paired Wilcoxon test).

**Fig. 2:**
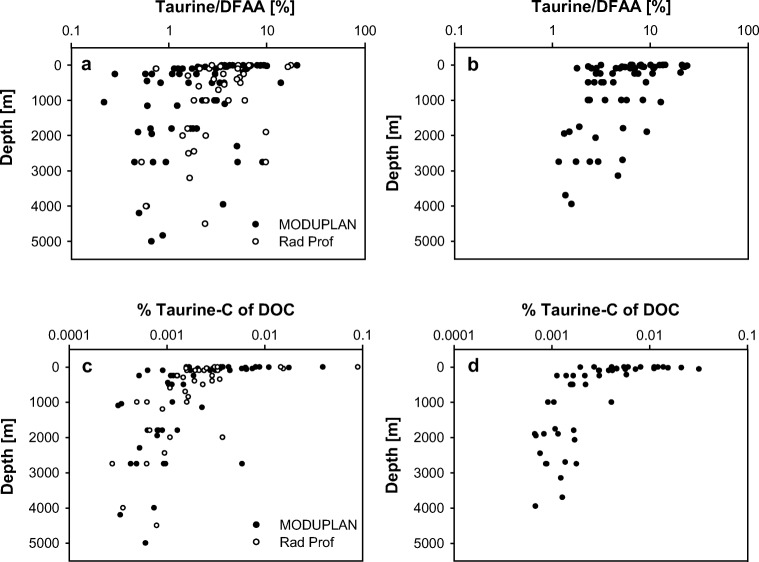
Percent contribution of taurine to DFAA (**a**, **b**), and % taurine-C of DOC (**c**, **d**) throughout the water column at the stations occupied during the MODUPLAN and the RadProf cruise in the North Atlantic Ocean. Data of transect 1 are shown in the left panels, and data of transect 2 are shown in the right panels

Dissolved free taurine concentrations (Fig. S5a, b) showed similar distribution patterns with depth in both transects. Taurine concentrations ranged from 0.2 to 16 nM in epipelagic waters (*n* = 73) and decreased with depth to 0.07–1.6 nM in bathypelagic waters (*n* = 48) or were below the detection limit (Table S1). In contrast to dissolved free Tau, dissolved free leucine concentrations showed no clear depth-dependent pattern ranging from 0.1 to 5.6 nM in the epipelagic (*n* = 72) and from 0.03 to 1.4 nM in the bathypelagic layer (*n* = 48) during both cruises (Fig. S5c, d). In both transects, the percentage of taurine to DFAA (Fig. 2a, b and Table S4) was highest in epipelagic waters (transect 1: 5.2 ± 4.3%, *n* = 43, transect 2: 9.3 ± 6.2%, *n* = 30), and decreased significantly with depth down to the bathypelagic realm (transect 1: 2.3 ± 2.6%, *n* = 32, transect 2: 3.5 ± 3.2%, *n* = 16; Kruskal–Wallis test followed by a Mann–Whitney *U* test, *p* < 0.05).

In contrast, the percentage of leucine to DFAA significantly increased with depth in both transects (Fig. S4c, d and Table S4; Kruskal–Wallis test followed by a Mann–Whitney *U* test, *p* < 0.05) with an average contribution of 1.8 ± 1.2% (*n* = 72) in the epipelagic waters, 2.1 ± 1.0% in the mesopelagic (*n* = 52), and 3.0 ± 2.2% in the bathypelagic (*n* = 49) waters.

Similar to the depth distribution of DOC (Fig. S6a, b), the contribution of taurine-C to the DOC pool (Fig. 2c, d) was highest in surface waters (0.008 ± 0.01%, *n* = 61, *p* < 0.05, Kruskal–Wallis test followed by a Mann–Whitney *U* test) and decreased with depth down to 0.001 ± 0.001% in bathypelagic waters (*n* = 42) in both transects. In contrast, the contribution of leucine-C to DOC (Fig. S6c, d) was fairly uniform throughout the water column (epipelagic: 0.004 ± 0.004%, *n* = 60; mesopelagic: 0.005 ± 0.007%, *n* = 39; bathypelagic: 0.005 ± 0.003%, *n* = 41).

### Taurine Assimilation and Respiration and Leucine Incorporation

Prokaryotic abundance (Fig. S7) ranged from 1.4 × 10^5^ to 1.2 × 10^6^ cells mL^−1^ (*n* = 73) in the epipelagic layers and decreased by 1 to 2 orders of magnitude with depth reaching 1.1 × 10^4^–5.8 × 10^4^ cells mL^−1^ in the bathypelagic layers (*n* = 50) with no discernible differences between the research cruises or transects. In transect 1, average prokaryotic taurine assimilation and respiration rates in the epipelagic waters amounted to 0.78 ± 0.75 nmol L^−1^ day^−1^ (*n* = 27) and 0.64 ± 0.62 nmol L^−1^ day^−1^ (*n* = 28), respectively (Fig. S8a). In transect 2, average assimilation and respiration rates in the epipelagic waters were 0.73 ± 0.50 nmol L^−1^ day^−1^ (*n* = 17) and 0.93 ± 0.83 nmol L^−1^ day^−1^ (*n* = 18), respectively (Fig. S8b). In transect 1, taurine assimilation decreased with depth by three orders of magnitude averaging 0.005 ± 0.004 nmol L^−1^ day^−1^ (*n* = 13) between 2000 and 5000 m depth while taurine respiration declined by two orders of magnitude down to the lower bathypelagic realm (0.06 ± 0.06 nmol L^−1^ day^−1^; 2000–5000 m; *n* = 12: Fig. S8a). In transect 2 (Fig. S8b), taurine assimilation and respiration declined by two orders of magnitude with depth reaching 0.01 ± 0.01 nmol L^−1^ day^−1^ (*n* = 8) and 0.02 ± 0.03 nmol L^−1^ day^−1^ (*n* = 9), respectively, in the lower bathypelagic realm (2000–4000 m). Generally, the decrease (slope) in assimilation and respiration rates with depth was remarkably similar in both transects (slopes obtained from data of transect 1 and transect 2 were not significantly different: *t* test *p* > 0.05; Fig. S8a, b). Taurine assimilation rates were positively related to the % contribution of taurine to DOC (*R*^2^ = 0.45, power regression, *p* < 0.0001), however, only weakly with % taurine of DFAA (*R*^2^ = 0.18, *p* < 0.0001).

Total taurine uptake (assimilation + respiration) was similar between the depth layers of the two transects (paired Wilcoxon test, *p* > 0.05). Total taurine uptake, however, was significantly higher in the epipelagic (1.5 ± 1.2 nmol L^−1^ day^−1^; *n* = 46) than in the meso- (0.1 ± 0.2 nmol L^−1^ day^−1^, *n* = 53) and the bathypelagic (0.05 ± 0.05 nmol L^−1^ day^−1^, *n* = 43) waters (*p* < 0.05, Kruskal–Wallis test followed by a Mann–Whitney *U* test) (data shown for taurine assimilation and respiration separately in Fig. S8a, b).

**Fig. 3:**
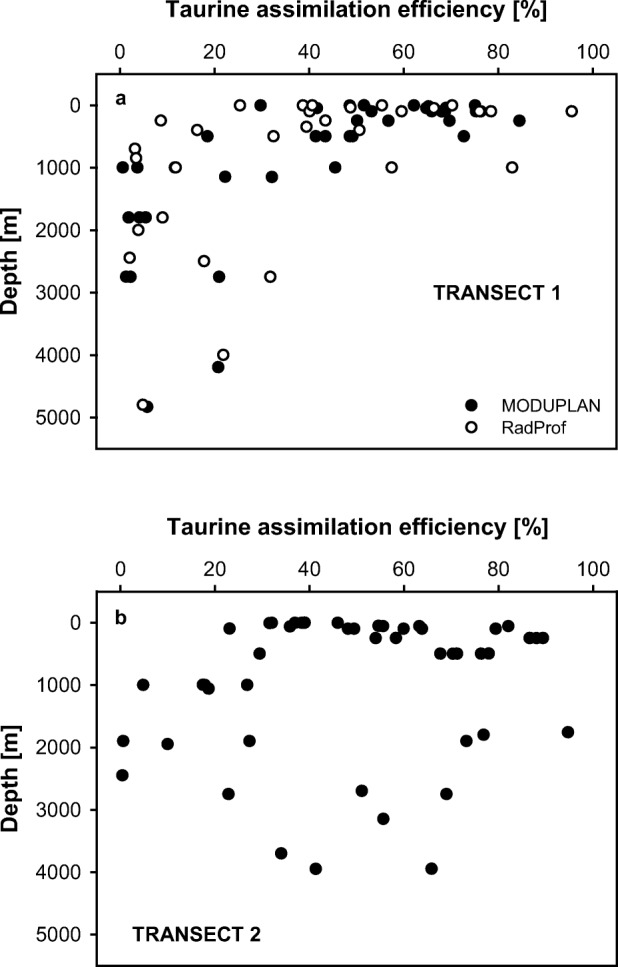
Depth profiles of taurine assimilation efficiency at transect 1 (**a**) and transect 2 (**b**)

The highest taurine assimilation efficiency was detected in the deep chlorophyll maximum layer of transect 1 (median 68.8%, Fig. 3a), located between 50 and 100 m depth and in the North Atlantic Central Water [53] located between 200 and 500 m of transect 2 (median 76.3%, Fig. 3b). However, no significant differences were observed within the depth layers between the two transects, except for the upper mesopelagic layer (200–500 m, paired Wilcoxon test, *p* < 0.05).

Leucine incorporation rates of the bulk prokaryotic community exhibited a similar depth-related trend as the bulk taurine assimilation (Fig. S8c, d). Leucine incorporation rates decreased from epipelagic waters (0.008–1.4 nmol L^−1^ day^−1^, *n* = 65), however, by up to five orders of magnitude to the bathypelagic zone (0.0007–6.9 pmol L^−1^ day^−1^; *n* = 46). Leucine incorporation rates showed a similar depth-related trend in both transects (Fig. S8c, d). In the epipelagic and mesopelagic waters, bulk leucine incorporation rates were on average 13.3 ± 9.5 and 8.3 ± 7.8 times lower than taurine assimilation, respectively. In the bathypelagic zone, however, leucine incorporation was on average 41.2 ± 61.6 times lower than taurine assimilation rates (Fig. S8). Leucine incorporation rates were negatively related to % contribution of leucine to DFAA (*R*^2^ = 0.30, power regression), while no relationship was obtained with % contribution of leucine to DOC (*R*^2^ = 0.02, power regression).

**Fig. 4:**
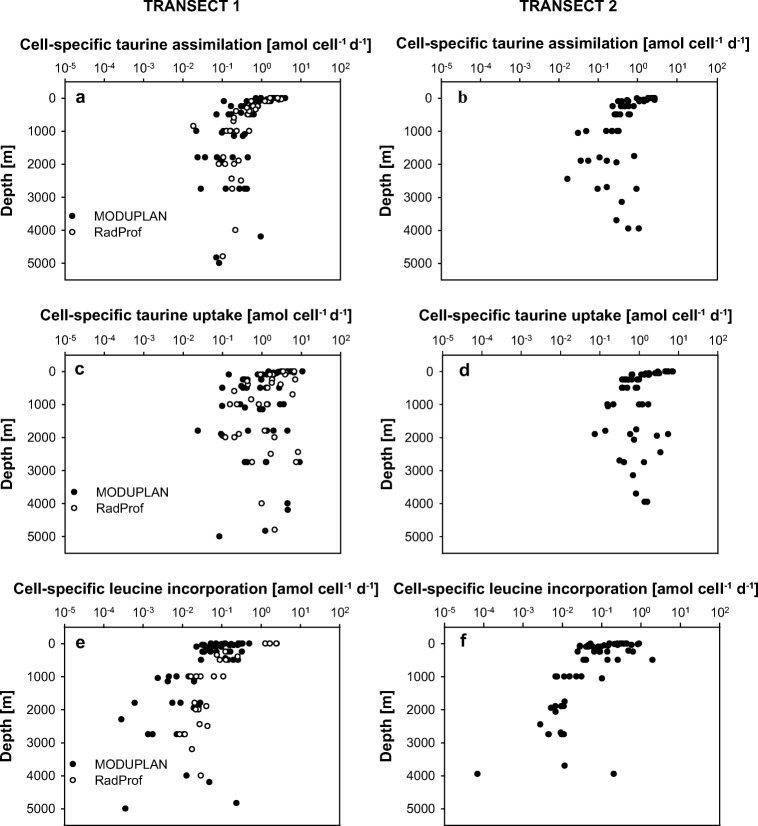
Cell-specific taurine assimilation (**a**, **b**) and uptake (**c**, **d**) rates and cell-specific leucine incorporation rates (**e**, **f**) throughout the water column at the stations occupied during the MODUPLAN and the RadProf cruise in the North Atlantic Ocean. Data of transect 1 are shown in the left and data of transect 2 in the right panels

Cell-specific taurine assimilation (Fig. 4a, b), as calculated based on the total prokaryotic abundance, ranged between 0.1 and 4.0 amol cell^−1^ day^−1^ (*n* = 44) in the epipelagic realm and decreased with depth only by 1–2 orders of magnitude (mesopelagic: 0.02–0.8 amol cell^−1^ day^−1^, *n* = 53; bathypelagic: 0.02–1.1 amol cell^−1^ day^−1^, *n* = 43). Cell-specific taurine uptake (assimilation + respiration) varied between 0.02 and 10.8 (amol cell^−1^ day^−1^; epipelagic: *n* = 46, mesopelagic *n* = 53, bathypelagic *n* = 43) and did not exhibit a clear depth-related pattern (Kruskal–Wallis test, *p* > 0.05; Fig. 4c, d). Also, no differences in cell-specific taurine uptake were observed between the two transects (paired Wilcoxon test, *p* > 0.05). The highest cell-specific leucine incorporation was also detected in the epipelagic layer (0.02–2.5 amol cell^−1^ day^−1^, *n* = 65) decreasing with depth and varying from 0.005 to 2.0 amol cell^−1^ day^−1^ in the mesopelagic (*n* = 48) and from 0.00007 to 0.2 amol cell^−1^ day^−1^ in the bathypelagic waters (*n* = 46) (Fig. 4e, f).

### Dissolved Free Taurine and Leucine Turnover Rates

**Fig. 5:**
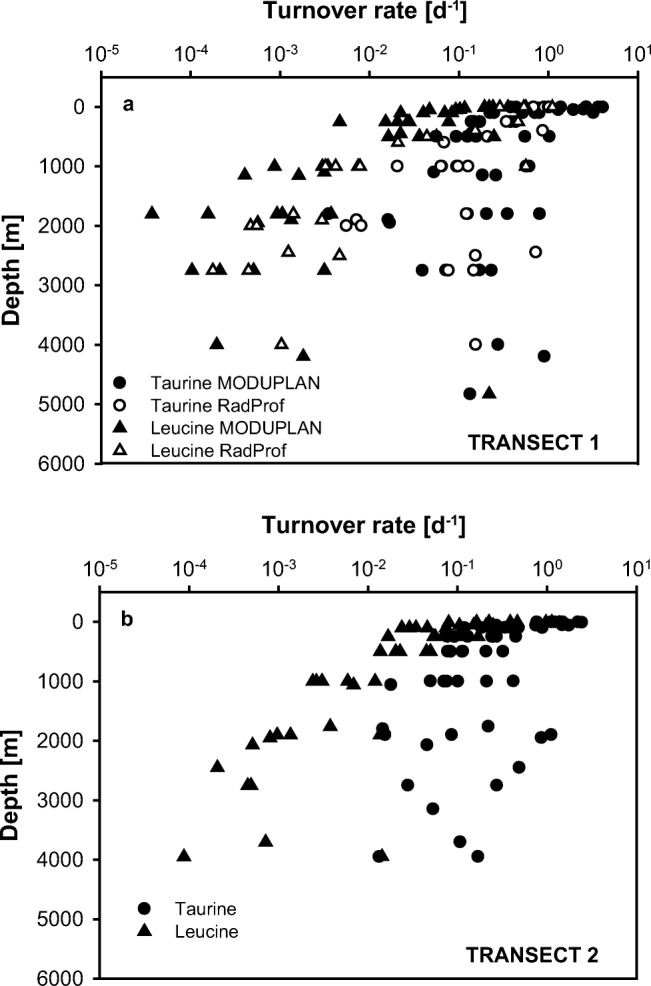
Turnover rates of taurine based on bulk taurine uptake (assimilation + respiration) rates and leucine based on bulk leucine incorporation rates. Depth profiles in **a** transect 1 and **b** transect 2

Taurine turnover rates (Fig. 5a, b) in the epipelagic realm ranged from 0.1 to 4.0 day^−1^ and were significantly higher than those in the meso- and bathypelagic realm (*p* < 0.05, Kruskal–Wallis test followed by a Mann–Whitney *U* test). Between 500 and 5000 m depth, taurine turnover rates were highly variable (0.004 to 1.1 day^−1^); however, the medians of the different water layers remained fairly stable.

Leucine turnover rates (Fig. 5a, b) were estimated based on the incorporation of ^3^H-leucine into prokaryotic protein with the assumption that leucine respiration is negligible [7, 54]. Hence, these estimates are likely conservative as this might not be always the case [55]. Moreover, the concentration of the radiolabeled leucine (5 nM) added to the samples is close to the ambient water leucine concentrations in the epipelagic but higher than leucine concentrations in deep waters which might result in increased leucine respiration [56]. Unfortunately, no data on leucine respiration in the deep ocean are available. Similar to taurine, leucine turnover rates declined from the epipelagic (ranging between 0.01 and 4.6 day^−1^) to the bathypelagic zone (0.00001 and 0.05 day^−1^). In contrast to taurine turnover, however, leucine turnover rates declined continuously with depth.

### Contribution of Taurine-C, -N, and -S to Prokaryotic Biomass Production

The contribution of taurine-C to prokaryotic biomass production was variable throughout the water column averaging 21 ± 15% in the epipelagic, 12 ± 12% in the mesopelagic, and 16 ± 16% in the bathypelagic realm (Table 1) albeit there were no significant differences detectable between the depth layers (*p* > 0.05, Kruskal–Wallis test). The potential contribution of taurine-N to the N-requirements of prokaryotes averaged 38 ± 34% in epipelagic waters, 26 ± 25% in mesopelagic, and 38 ± 34% in bathypelagic waters. The potential contribution of taurine-S to S-requirements was always > 100% (259 ± 219% in epipelagic, 169 ± 165% in mesopelagic, and 250 ± 218% in bathypelagic waters).

**Table 1:** Contribution of taurine-C to heterotrophic prokaryotic biomass production measured via leucine incorporation [%] in the different depth layers. SD, standard deviation; *n*, number of samples

Depth layer	Depth range [m]	Mean [%] SD		*n*
Epipelagic	0–50	23	16	18
	5–100	19	15	13
Mesopelagic	200–500	9	8	29
	500–1000	19	16	16
Bathypelagic	1000–2000	19	18	16
	2000–5000	14	14	16

### Contribution of Different Prokaryotic Groups to Taurine Uptake

**Fig. 6:**
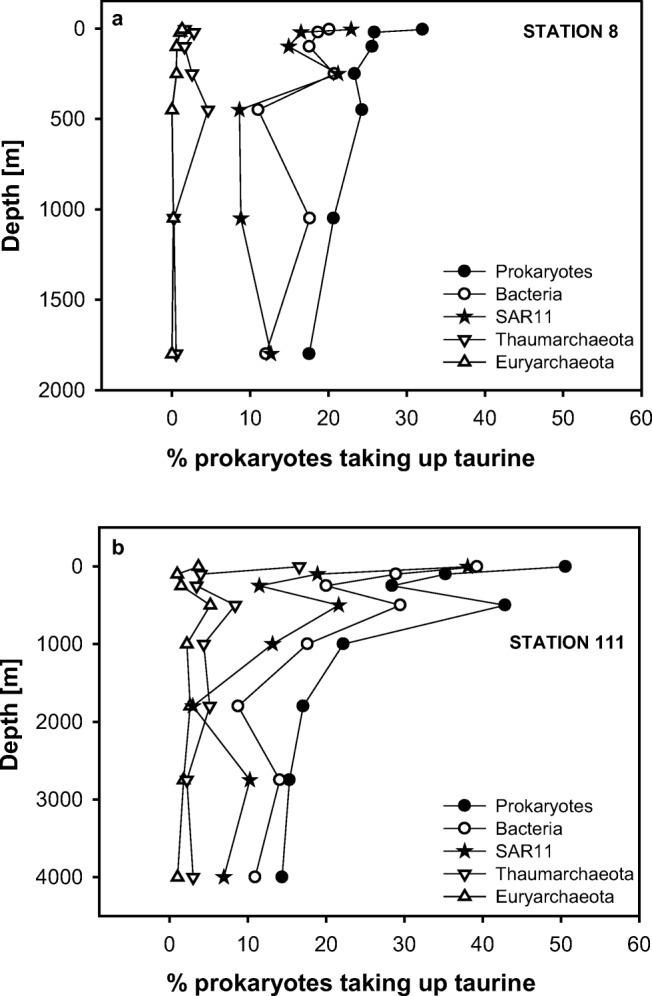
Percentage of total prokaryotic cells (DAPI-stained cells), Bacteria, SAR11, Thaumarchaeota, and Euryarchaeota taking up taurine in relation to the abundance of prokaryotes (DAPI-stained cells) throughout the water column at station 8 (**a**) and station 111 (**b**) of the MODUPLAN cruise

Based on MICRO-CARD-FISH analysis, the contribution of SAR11 to the total prokaryotic cells (DAPI-stained cells) ranged between 14 and 88%, while Thaumarchaeota contributed between 4 and 31% and Euryarchaeota were always below 15% (Table 2, Fig. S9). About 32% and 50% of the total prokaryotic cells were taking up taurine in the epipelagic waters at station 8 (continental slope site) and station 111 (oceanic site), respectively (Fig. 6). The contribution of prokaryotic cells taking up taurine decreased with depth to ~ 18% at 1800 m depth at station 8 (Fig. 6a) and to ~ 14% at 4000 m at station 111 (Fig. 6b). Bacteria taking up taurine amounted to 11–20% at station 8 (Fig. 6a) and 18–39% at station 111 (Fig. 6b) of the total prokaryotic abundance in epi- and mesopelagic waters. In bathypelagic waters, Bacteria taking up taurine accounted for 9 to 11% of the prokaryotic abundance (Fig. 6a, b). Members of the SAR11 cluster taking up taurine amounted up to ~ 20% (station 8) and 38% (station 111) of the prokaryotic cells in epi- and upper mesopelagic waters and decreased to ~ 10% and 13% at 1000 m depth at station 8 and station 111, respectively (Fig. 6a, b). Thaumarchaeota taking up taurine contributed 0.5 to 5% (station 8) and 3 to 17% (station 111) to the total prokaryotic abundance, while Euryarchaeota taking up taurine always contributed ≤ 1% at station 8 and ≤ 5% at station 111 to the total prokaryotic abundance (Fig. 6a, b).

**Table 2:** Contribution of bacterial and archaeal groups (% of total prokaryotic cells) through the water column from stations 8 and 111, as determined with CARD-FISH. Thaum, Thaumarchaeota cells; Eury, Euryarchaeota cells

	Depth (m)	Bacteria	SAR11	Thaum	Eury
Station 8	1800	72.0	70.3	4.0	0.0
	1050	93.6	69.6	0.7	0.6
	450	47.8	30.5	13.7	0.0
	250	73.8	88.1	12.6	3.0
	100	65.5	58.5	6.5	2.3
	21	74.2	66.0	10.4	3.5
	5	70.6	69.3	5.1	3.7
Station 111	4000	65.6	15.9	18.2	3.7
	2752	62.5	60.0	10.0	8.3
	1799	57.3	13.5	11.7	12.0
	1002	71.3	66.5	23.7	8.0
	500	82.6	38.0	16.6	15.0
	252	67.3	36.1	12.9	5.8
	102	74.9	46.2	13.3	4.4
	8	69.0	58.1	31.4	8.6

The contribution of Bacteria to the total number of prokaryotes taking up taurine was slightly higher than the bacterial contribution to the total prokaryotic abundance (Fig. S9a, b) in deep waters (500–4000 m) at both stations. In the upper mesopelagic and epipelagic zone, however, the contribution of Bacteria taking up taurine was almost always proportional to their contribution to prokaryotic abundance. Cells of the SAR11 cluster taking up taurine contributed proportionally or a slightly higher fraction to the prokaryotic community taking up taurine than to the total prokaryotic abundance, except at 500 m depth at station 8 and at 4000 m depth at station 111, where their contribution was lower (Fig. S9a, b). Archaea (Thaumarchaeota and Euryarchaeota) contributed proportionally to the prokaryotic community taking up taurine and to the prokaryotic abundance (Fig. S9a, b).

Cell-specific taurine assimilation rates of prokaryotes taking up taurine (i.e., surrounded by a silver grain halo) were calculated based on the bulk taurine assimilation rate and the abundance of prokaryotes taking up taurine (Fig. S9c). Cell-specific taurine assimilation was highest in epipelagic waters (7 amol cell^−1^ day^−1^) and decreased exponentially with depth to 0.13 amol cell^−1^ day^−1^ at 1800 m at station 8 (Fig. S9c). Cell-specific taurine assimilation (Fig. S9c) at station 111 was also highest in epipelagic waters (1.4–1.7 amol cell^−1^ day^−1^), however, lower than at the continental slope station (station 8). In the upper mesopelagic waters, the cell-specific taurine assimilation rate was similar at both stations (1.2 to 1.6 amol cell^−1^ day^−1^). Cell-specific taurine assimilation showed a slight increase at ~ 3000 m at station 111 (2.8 amol cell^−1^ day^−1^), whereas taurine assimilation was not detectable at 4000 m depth at this station.

### Global Distribution of Prokaryotic Taurine Transporters in the Ocean

Prokaryotes harboring taurine transporters are ubiquitous in the global ocean (Fig. S10). There are some variations in the fraction of prokaryotes encoding taurine transporters in different oceanographic regions (Fig. S10), however, with highest contributions in the Antarctic Province and lowest in the Red Sea and the Indian Monsoon Gyres. Most prominently, the contribution of prokaryotes encoding taurine transporters to the total prokaryotic community significantly increased from epipelagic (median 0.22 in subsurface waters, 0.19 in the deep chlorophyll maximum) to mesopelagic waters (0.46) (ANOVA on ranks *p* < 0.001), as indicated by the ratio taurine transporters: *rec*A + *rad*A (Figs. S10, S11).

## Discussion

Prokaryotes dominate the biomass of marine ecosystems [57], particularly in the deep ocean [58]. However, the sources of C and energy sustaining the heterotrophic prokaryotic activity throughout the water column remain largely enigmatic. Whereas in the surface ocean, phytoplankton release organic compounds, such as DFAA, proteins, or carbohydrates that prokaryotes can utilize [59], the organic compounds and sources available in deeper waters are less well known. Marine snow and zooplankton, through their vertical migration and fecal pellet production, contribute freshly produced organic matter to deeper layers of the ocean, including taurine [11]. However, the contribution and significance of this organic compound to the ocean prokaryotic production have not been evaluated yet.

DFAA concentrations in the study area exhibited the commonly reported depth-related pattern as previously reported for open oceans [60, 61]. The differences in the distribution pattern of dissolved free taurine and leucine (Fig. S5; and Table S4) and of other DFAA species with depth might indicate differences in their formation and/or utilization. The main sources of taurine in epipelagic waters are most likely phytoplankton [14, 15] and mesozooplankton [10, 11]. Mesozooplankton biomass and grazing pressure by the metazoan food web are highest in the epipelagic layer and decrease with depth [62, 63], potentially supporting the elevated taurine concentrations found in surface waters. The extent to which taurine is generated by deep-sea mesozooplankton remains to be determined. It is likely, however, that meso- and bathypelagic mesozooplankton produce taurine to counteract hydrostatic pressure [16]. Other sources of taurine in the deep sea might be fecal pellets and zooplankton carcasses as summarized in Clifford et al. [11]. In contrast to taurine, free leucine is released in significant amounts mainly by phytoplankton in the epipelagic layer [64]. Leucine release by crustacean zooplankton was also observed occasionally at the study site and the Gulf of Alaska (0.2–5.9 μmol g^−1^ C-biomass h^−1^), but at lower rates than taurine (0.5–11.8 μmol g^−1^ C-biomass h^−1^) and glycine (0.4–57.4 μmol g^−1^ C-biomass h^−1^) [11]. A similar trend was found for zooplankton collected off Sapelo Island (Webb and Johannes, 1968). Leucine together with aspartic acid, glycine, and serine was also found to be the dominant DFAA species in copepod fecal pellets [65].

**Fig. 7:**
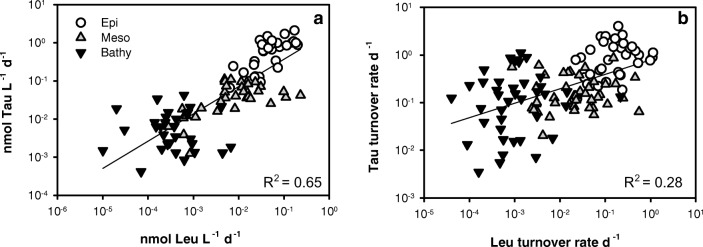
Taurine assimilation versus leucine incorporation (**a**) and taurine turnover versus leucine turnover (**b**) in the Atlantic Ocean off the Iberian Peninsula

Membrane-bound transporter proteins for taurine are frequently found in marine prokaryotes suggesting that taurine is widely utilized as a substrate [29, 66], not only in the epipelagic waters but also in the meso- and bathypelagic realm [59] (Fig. S10). Moreover, a recent metagenomic study also suggested that taurine is an important substrate for zooplankton-associated prokaryotes [67]. The average uptake rates of taurine in the epipelagic (1.23 nmol L^−1^ day^−1^, range 0.16–3.31 nmol L^−1^ day^−1^) closely match the estimated release rates of zooplankton (1.1–2.6 nmol taurine L^−1^ day^−1^) [11], suggesting a balance between production and utilization of taurine. Unfortunately, bulk taurine release could not be estimated for meso- and bathypelagic waters, due to the lack of zooplankton abundance and biomass data. However, the general increase in the contribution of prokaryotes encoding taurine transporters (Figs. S10 and S11) from epipelagic to mesopelagic waters, the increase in taurine transporters with depth [59], and the increase in the relative abundance of cells taking up taurine (Fig. 6) with depth indicate that zooplankton are likely a major source of taurine for prokaryotes inhabiting the mesopelagic realm. About 80% of the variation of taurine assimilation in the water column is explained by depth in both transects (Fig. S8a, b, Table S5), which is similar to the depth dependency of leucine incorporation (Fig. S8c, d, Table S5). Accordingly, leucine incorporation explains 65% of the variation of taurine assimilation (Fig. 7a). The decrease of leucine incorporation with depth is remarkably similar to that reported for the Pacific and the Atlantic Ocean [68, 69].

The taurine assimilation efficiency of epipelagic prokaryotes ranged from 41 to 69% (Fig. 3) and was therefore similar to the average assimilation efficiency of DFAA of 65% found in coastal water [70]. Interestingly, taurine assimilation efficiency (Fig. 3) was highest in the lower epipelagic layer in transect 1 and in the upper mesopelagic in transect 2, coinciding with the depth distribution of the main taurine producers: phytoplankton and mesozooplankton [71, 72]. The taurine turnover rates obtained in this study (0.12 to 4.0 day^−1^, Fig. 5) are similar to the taurine turnover rates (1.0–2.3 day^−1^) obtained by determining mesozooplankton release rates in combination with mesozooplankton abundance data of the North Atlantic Ocean [11]. Consequently, the low concentrations of dissolved free taurine (Fig. S5a, b) throughout the water column and its fast turnover rates suggest a tight coupling between release processes and prokaryotic consumption, as shown for DFAA [7, 37]. Taurine turnover rates in the individual depth layers are about one order of magnitude higher than leucine turnover rates in the epipelagic and mesopelagic waters and two orders of magnitude higher in the bathypelagic waters (Figs. 5 and 7b).

Taurine-C assimilation contributed to heterotrophic prokaryotic biomass production on average 21 ± 15% in the epipelagic layer and 16 ± 16% in the bathypelagic layers (Table 1). These estimates should be considered with caution due to the large variability in the conversion factor from prokaryotic leucine incorporation to C-biomass production, ranging between 0.02 and 1.92 kg C mol^−1^ Leu [73–75]. The 20-fold variation in conversion factors can result in large over- or underestimation of the actual biomass production based on leucine [73]. The large variation in the conversion factors together with possible higher respiration rates of leucine in deep waters associated with the use of radiolabelled leucine at higher than environmental concentrations [56] could help explain the low correlation between turnover rates of leucine and taurine (Fig. 7b). However, the taurine assimilation and turnover rates are within the range of those of DFAA [7, 37]. This, together with the moderate decrease in taurine respiration with depth as compared to the assimilation (Fig. S8a, b), indicates that taurine represents an important C and energy source for heterotrophic marine prokaryotes throughout the oceanic water column.

Members of the SAR11 clade were abundant throughout the water column in transect 1, particularly in the euphotic layer where they accounted for approximately 80% of the community, as revealed by next generation sequencing [42] and CARD-FISH analysis (Fig. S9). SAR11 also accounted for a large fraction of Bacteria taking up taurine, especially in the epipelagic layers (Fig. 6). The importance of taurine as a favorable C-source for SAR11 members is well documented [76, 77]. Taurine-S metabolism by SAR11 and other heterotrophic prokaryotes has been proposed to be important in oxygen minimum zones [28, 78] and in the deep ocean [27]. Thus, taurine may serve as a significant S-source for members of the SAR11 clade and other heterotrophic bacteria lacking the ability to take up sulfate, especially in the deep ocean [32]. The increase with depth in the contribution of TauD (alpha-ketoglutarate-dependent taurine dioxygenase; [59]), a gene used to break down taurine as a source of S especially under stress conditions [79], supports this notion. This increase in TauD agrees with the lower availability of other organic S-compounds, such as dimethylsulfoniopropionate, produced in the epipelagic layer by phytoplankton and reported to match the S-demands of SAR11 in the surface waters [32]. Our estimates show that taurine-S could also potentially match the S-requirements of prokaryotes, and thus, it could replace other organic S-substrates, particularly in the dark ocean. Based on genomic evidence, mixotrophic growth on taurine has been proposed for some isolates of Thaumarchaeota [80] but has not been experimentally shown yet. In our study, Thaumarchaeota as well as Euryarchaeota were able to take up taurine, especially in the epi- and upper mesopelagic zone (0–500 m depth). However, their contribution to the total prokaryotic community taking up taurine was minor (Fig. 6).

Taken together, our results show that taurine can sustain an important fraction of heterotrophic and/or mixotrophic prokaryotic production in the world’s oceans. Taurine is particularly important for meso- and bathypelagic prokaryotes, due to the release of taurine by zooplankton and other metazoans at these otherwise nutrient-limited environments. Taurine can sustain a significant fraction of heterotrophic prokaryotic C-biomass production, but can also potentially support a high proportion of the N- and S-demands. Given that the elemental composition of dissolved organic matter changes with depth, with increasing C:N [81], and C:S ratios [82], the release of compounds such as taurine at greater depth by zooplankton represents a fresh and nutritionally valuable source of C, N, S, and energy for deep-sea prokaryotes.

## Electronic Supplementary Material

ESM 1 (PDF 20092 kb)

ESM 2 (XLSX 27 kb)
