# Supplementary material for: Taurine Is a Major Carbon and Energy Source for Marine Prokaryotes in the North Atlantic Ocean off the Iberian Peninsula
Source: Microb Ecol. 2019 Jan 21;78(2):299–312. doi: 10.1007/s00248-019-01320-y (PMC6647121; doi:10.1007/s00248-019-01320-y)
Supplement: Supplementary file 1 — (PDF 20092 kb) [file 248_2019_1320_MOESM1_ESM.pdf]

Supplementary Information:

Article title: Taurine is a major carbon and energy source for marine prokaryotes in the North Atlantic Ocean off the Iberian Peninsula

Journal name: Microbial Ecology

Author names: Elisabeth L. Clifford[a], Marta M. Varela[b], Daniele De Corte[c], Antonio Bode[b], Victor Ortiz[a], Gerhard J. Herndl[a, d], Eva Sintes[a,e*]

[a] University of Vienna, Department of Limnology and Bio-Oceanography, Center of Functional Ecology, Althanstrasse 14, 1090 Vienna, Austria

[b] Instituto Español de Oceanografía (IEO), Centro Oceanográfico de A Coruña, Apdo 130, 15080 A Coruña, Spain

[c] Research and Development Center for Marine Biosciences, Japan Agency for Marine-Earth Science and Technology (JAMSTEC), Natushima 2-15, Yokosuka, Kanagawa 237-0061, Japan

[d] Royal Netherlands Institute for Sea Research (NIOZ), Department of Marine Microbiology and Biogeochemistry, Utrecht University, PO Box 59, 1790 AB Den Burg, The Netherlands

[e] Instituto Español de Oceanografía (IEO), Centro Oceanográfico de Baleares, Moll de Ponent s/n, 07015 Palma de Mallorca, Spain

*Address and phone number for correspondence: eva.sintes@univie.ac.at; +34-971133720

Table S1. Parameters to validate the HPLC method.

**DFAA**[a]	**Linearity [nM]**	**R^2**	**LOD/LOQ [nM]**	**Recovery/RSD [*n* = 5, %]**		
				1nM	5nM	10nM
**ASP**	0.02-100	0.999	0.02/0.06	90/6.3	104/1.6	101/0.5
**GLU**	0.01-100	0.999	0.01/0.04	100/8.2	104/4.1	100/0.1
**ASN**	0.01-100	0.999	0.01/0.04	98/0.7	105/3.6	95/0.5
**SER**	0.02-100	0.999	0.02/0.06	104/2.3	86.7/5.7	87/22
**GLN**	0.04-100	0.999	0.04/0.12	95/1.4	106/3.6	101/1.1
**HIS**	0.06-100	0.999	0.06/0.18	97/1.4	108/4.9	102/1.4
**GLY**	0.01-100	0.998	0.01/0.03	97/2.0	96/2.5	98.8/1.1
**ARG**	0.01-100	0.999	0.01/0.03	103/2.0	109/6.5	101/1.1
**THR**	0.06-100	0.999	0.06/0.20	96/3.7	108/7.2	100/1.0
**ALA**	0.01-100	0.999	0.01/0.05	102/0.9	100/2.9	100/5.4
**TAU**	0.01-100	0.998	0.01/0.05	99/0.5	100/0.2	101/2.9
**GABA**	0.01-100	0.999	0.01/0.05	95/3.6	89/3.6	100/0.1
**TYR**	0.04-100	0.999	0.04/0.12	97/5.7	80/3.6	100/0.32
**VAL/MET**	0.03-100	0.999	0.03/0.09	102/4.2	102/6.4	100/7.8
**TRP**	0.02-100	0.998	0.02/0.06	101/3.2	102/0.9	99/3.5
**PHE**	0.01-100	0.997	0.01/0.04	98/5.2	105/1.9	90/16.4
**ILE**	0.01-100	0.999	0.01/0.03	100/4.1	103/2.8	99/6.2
**LEU**	0.01-100	0.999	0.01/0.03	102/1.7	106/2.3	104/10.3
**LYS**	0.03-100	0.999	0.03/0.10	100/0.9	107/2.7	102/1.9

[a] DFAA species abbreviations: R^2, correlation coefficient; LOD, limit of detection; LOQ, limit of quantification; RSD, relative standard deviation; Asp, Aspartic acid; Glu, Glutamic acid; Asn, Asparagine; Ser, Serine; Gln, Glutamine; His, Histine; Gly, Glycine; Arg, Arginine; Thr, Threonine; Ala, Alanine; Tau, Taurine; GABA, gamma aminobutyric acid; Tyr, Tyrosine; Met, Methionine; Val, Valine; Trp, Tryptophan; Phe, Phenylalanine; Ile, Isoleucine; Leu, Leucine; Lys, Lysine

Table S2. Probes and hybridization conditions used for MICRO-CARD-FISH analyses. Hyb. Temp: Hybridization temperature.

Probe name	Sequence (5' – 3')	Target group	Formamide [%]	Hyb. Temp. [°C]	Reference
NON338	ACTCCTACGGGAGGCAGC	negative control probe	55	35	Amann et al. (1995) [1]
EUB 338 I	GCTGCCTCCCGTAGGAGT	most Bacteria	55	35	Amann et al. (1990) [2]
EUB 338 II	GCAGCCACCCGTAGGTGT	Planctomycetales	55	35	Daims et al. (1999) [3]
EUB 338 III	GCTGCCACCCGTAGGTGT	Verrucomicrobiales, Chloroflexi	55	35	Daims et al. (1999) [3]
SAR11-152R	ATTAGCACAAGTTTCCYCGTGT	SAR 11 cluster	45	35	Morris et al. (2002) [4]
SAR11-441R	TACAGTCATTTTCTTCCCCGAC	SAR 11 cluster	45	35	Morris et al. (2002) [4]
SAR11-542R	TCCGAACTACGCTAGGTC	SAR 11 cluster	45	35	Morris et al. (2002) [4]
SAR11-732R	GTCAGTAATGATCCAGAAAGYTG	SAR 11 cluster	45	35	Morris et al. (2002) [4]
CREN 537	TGACCATTGAGGTGCTG	Thaumarchaeota	20	35	Teira et al. (2004) [5]
CREN 554	TTAGGCCCAATAATCMTCCT	Thaumarchaeota	20	35	Woebken (2007) [6]
EURY 806	CACAGCGTTTACACCTAG	Euryarchaeota	20	35	Teira et al. (2004) [5]

Table S4a. Dissolved free amino acids (DFAA) throughout the water column at the stations occupied during the MODUPLAN cruise in the North Atlantic Ocean. The total DFAA and the percentages of dissolved taurine and leucine to the total DFAA [%] are also indicated. Concentrations between the detection and quantification limits are marked in grey. Abbreviations: Asp, Aspartic acid; Glu, Glutamic acid; Asn, Asparagine; Ser, Serine; Gln, Glutamine; His, Histine; Gly, Glycine; Arg, Arginine; Thr, Threonine; Ala, Alanine; Tau, Taurine; GABA, gamma aminobutyric acid; Tyr, Tyrosine; Met, Methionine; Val, Valine; Trp, Tryptophan; Phe, Phenylalanine; Ile, Isoleucine; Leu, Leucine; Lys, Lysine.

Station	Depth	ASP	GLU	ASN	SER	GLN	HIS	GLY	ARG	THR	ALA	TAU	GABA	TYR	VAL/MET	TRP	PHE	ILE	LEU	LYS	total DFAA	Tau/DFAA [%]	Leu/DFAA [%]
5	5	2.45	6.13	8.42	4.28	6.64	7.00	4.66	1.79	2.17	11.36	15.66	1.48	0.45	1.28	-	0.42	1.24	0.78	0.98	77.18	20.29	1.01
5	9	1.16	3.25	6.50	7.95	2.32	1.95	7.93	2.02	0.78	7.39	4.39	0.24	0.52	1.05	-	0.50	1.39	0.91	1.08	51.33	8.56	1.76
5	40	0.89	2.48	5.02	9.78	0.46	1.37	5.82	1.86	0.25	3.87	2.04	0.05	0.42	0.83	-	0.84	2.42	0.76	0.96	40.12	5.09	1.88
5	100	1.95	2.66	4.29	5.56	0.74	1.36	2.82	0.86	0.08	1.41	0.27	0.05	0.17	0.37	0.20	0.50	0.19	0.24	-	23.71	1.13	1.02
5	202	1.56	2.88	4.05	6.51	0.45	0.93	3.31	0.25	1.10	2.13	1.59	0.54	0.30	0.68	-	0.10	0.30	0.58	0.54	27.80	5.73	2.07
8	3	1.54	2.97	5.89	7.38	1.47	1.94	4.70	1.50	0.31	2.35	1.44	0.83	-	0.54	0.01	0.24	0.26	0.48	0.62	34.48	4.17	1.39
8	21	1.30	3.57	5.39	8.88	1.08	1.10	2.82	1.60	0.40	3.35	2.39	0.10	0.41	0.67	-	0.13	0.71	0.71	0.87	35.49	6.74	1.99
8	100	2.93	6.82	4.65	13.59	0.94	2.51	4.05	2.47	0.13	4.25	0.67	0.13	0.48	0.62	0.29	0.41	0.56	1.25	1.77	48.52	1.38	2.58
8	250	2.82	10.70	4.63	9.95	4.11	10.86	15.07	8.09	0.38	13.28	0.54	0.08	1.58	2.14	0.50	1.31	1.84	2.14	4.76	94.80	0.57	2.26
8	450	2.65	5.89	2.48	13.35	0.26	1.75	7.16	2.05	0.10	3.82	0.26	0.79	0.43	0.58	-	0.31	0.52	0.79	1.14	44.32	0.60	1.78
8	1050	1.70	4.10	2.48	11.17	0.65	1.95	5.82	1.61	0.05	3.19	0.08	0.73	-	0.46	0.18	0.32	0.39	1.21	2.71	38.82	0.22	3.12
8	1800	1.49	2.64	1.15	6.74	0.29	0.82	3.84	1.15	-	-	0.25	-	-	2.57	0.17	0.01	0.71	0.77	0.50	23.09	1.06	3.33
11	5	1.61	1.83	6.03	2.93	0.69	0.29	1.81	0.37	0.15	0.66	0.63	0.50	-	0.17	-	-	0.19	0.24	0.14	18.23	3.45	1.29
11	45	1.64	2.64	4.58	3.30	0.18	0.66	2.50	0.62	0.12	1.17	0.74	0.55	-	0.29	-	-	0.36	0.31	0.25	19.91	3.74	1.53
11	100	1.52	1.90	4.41	2.93	0.28	0.42	1.96	0.17	0.25	0.87	0.50	-	-	0.15	0.02	0.14	0.03	0.30	0.28	16.14	3.12	1.85
11	250	2.96	5.53	3.75	17.25	0.48	3.55	6.90	0.54	8.55	1.55	0.18	4.95	1.23	0.95	0.85	0.87	0.77	0.87	1.31	63.02	0.28	1.38
11	500	1.32	1.53	2.92	2.24	0.26	0.29	1.15	0.37	0.06	0.45	0.41	0.45	-	0.16	0.03	-	0.49	0.22	0.10	12.43	3.28	1.75
11	1150	1.00	2.22	2.12	6.99	0.22	0.86	4.17	0.63	0.26	1.89	0.28	-	-	0.66	-	0.06	0.24	0.70	1.08	23.39	1.21	2.99
11	1900	1.43	2.39	2.29	5.65	0.18	0.76	3.45	0.74	0.12	19.29	0.19	-	-	0.46	-	0.26	0.20	0.75	1.64	39.79	0.48	1.89
11	2750	1.89	3.68	1.73	7.37	0.20	0.95	4.41	0.93	0.11	2.01	0.12	-	0.23	0.58	-	0.10	0.55	0.53	0.72	26.13	0.44	2.05
14	5	1.21	2.67	4.98	5.35	0.64	0.44	4.35	1.41	0.59	2.57	2.56	0.08	0.28	0.92	-	0.13	0.20	0.54	0.53	29.44	8.70	1.82
14	50	1.22	3.38	4.14	10.92	0.88	2.03	5.05	0.74	1.93	5.43	0.80	0.03	0.41	1.12	0.44	0.27	0.37	0.65	0.74	40.55	1.97	1.60
14	101	0.97	1.60	3.91	5.92	0.67	0.88	3.39	1.24	0.04	1.60	0.31	-	-	0.95	0.50	0.61	1.48	0.43	0.46	24.95	1.23	1.71
14	250	1.51	0.85	3.16	1.81	0.27	0.25	0.37	0.44	0.41	0.59	0.32	0.03	0.02	0.03	-	-	0.31	0.20	0.13	10.70	2.95	1.82
14	500	0.91	1.16	2.36	2.63	0.52	0.76	1.38	0.51	0.11	0.67	0.56	-	0.05	0.24	-	1.61	1.35	0.25	0.25	15.32	3.65	1.62
14	1150	1.04	1.72	2.42	5.00	0.29	0.11	3.02	0.83	-	1.71	0.18	7.54	0.31	1.29	-	2.60	0.22	0.48	0.59	29.33	0.60	1.62
14	1950	1.29	2.79	2.08	9.40	0.92	0.40	4.89	2.09	0.21	3.05	0.21	0.96	-	0.80	0.14	0.15	0.50	0.83	1.10	31.82	0.67	2.60
14	2750	1.40	2.77	5.86	3.44	0.98	0.26	2.13	0.62	0.27	1.36	1.10	0.19	0.12	0.30	-	0.06	0.54	0.32	0.26	22.00	5.02	1.46

Station	Depth	ASP	GLU	ASN	SER	GLN	HIS	GLY	ARG	THR	ALA	TAU	GABA	TYR	VAL/MET	TRP	PHE	ILE	LEU	LYS	total DFAA	Tau/DFAA [%]	Leu/DFAA [%]
**16**	5	1.31	1.84	4.77	2.65	0.31	0.77	2.51	0.46	0.19	1.02	1.12	0.08	0.15	0.41	0.51	0.54	0.05	0.35	0.44	19.49	5.75	1.80
**16**	50	1.93	3.33	5.28	3.33	0.92	1.38	2.67	0.23	0.09	1.17	1.58	0.01	0.12	0.34	0.09	0.54	0.10	0.56	0.81	24.50	6.45	2.28
**16**	100	1.43	2.11	5.82	1.33	0.58	0.95	2.07	0.38	0.06	1.37	0.38	0.05	0.03	0.79	0.09	0.22	0.51	0.33	0.43	18.91	1.99	1.73
**16**	250	2.45	3.92	8.62	2.35	0.28	1.14	4.26	2.05	0.24	2.32	0.39	0.03	0.20	0.76	-	0.29	0.49	0.56	0.41	30.75	1.27	1.83
**16**	500	1.40	1.20	2.12	2.18	0.23	-	1.15	0.15	0.02	0.44	0.08	0.41	-	0.15	0.36	0.12	-	0.24	0.01	10.24	0.82	2.37
**16**	1100	1.39	1.17	2.84	1.65	0.18	0.29	1.08	0.15	0.01	0.50	0.40	0.08	-	0.27	-	-	0.36	0.29	0.22	10.89	3.71	2.64
**16**	1800	1.17	2.14	2.38	5.74	0.21	0.26	4.84	1.21	0.07	1.75	0.15	0.00	0.17	1.50	0.34	0.35	0.21	-	0.51	23.01	0.66	-
**16**	2300	1.25	2.11	5.67	5.41	0.99	1.31	3.34	1.01	0.44	2.04	1.39	0.20	0.30	0.47	-	0.23	0.92	0.48	0.50	28.05	4.95	1.72
**16**	3950	1.47	2.35	5.91	10.08	0.70	1.48	10.42	3.82	0.74	4.20	1.62	-	0.48	0.71	-	-	0.37	0.60	0.66	45.62	3.56	1.32
**103**	5	1.64	2.43	6.24	2.76	1.90	0.60	3.71	0.74	0.66	3.08	2.87	4.37	0.30	0.97	-	2.90	0.35	0.40	0.16	36.08	7.96	1.11
**103**	20	1.28	3.69	6.02	7.23	1.28	1.31	8.20	2.30	1.27	8.47	5.56	0.11	0.94	0.41	0.17	1.11	1.50	2.62	2.25	55.70	9.97	4.71
**103**	45	1.42	2.22	4.22	3.17	0.75	1.16	2.97	-	0.42	1.19	1.02	0.09	0.12	0.61	1.29	1.08	3.90	0.47	0.19	26.28	3.88	1.78
**103**	70	2.95	10.61	4.83	30.77	0.98	4.67	18.53	1.12	4.83	10.07	2.26	3.69	2.19	1.66	0.78	2.28	1.52	1.85	2.35	107.95	2.10	1.72
**108**	3	2.09	2.63	5.85	2.00	0.48	0.77	0.92	0.14	0.28	0.69	0.83	0.11	0.06	0.24	0.11	0.06	0.77	0.24	0.34	18.61	4.47	1.31
**108**	5	1.92	2.09	5.80	1.82	1.39	0.61	0.92	0.19	0.18	0.58	0.66	0.81	0.04	0.17	0.33	0.24	-	0.14	0.04	17.92	3.70	0.76
**108**	50	2.06	4.66	5.41	15.72	0.78	2.46	8.86	3.02	0.24	5.44	1.31	0.11	0.61	1.19	0.59	0.85	0.68	0.87	1.16	56.02	2.34	1.55
**108**	100	1.48	1.81	4.37	4.07	0.92	0.76	2.41	1.14	0.04	1.85	0.23	7.82	0.13	0.61	-	1.18	1.71	0.46	0.07	31.07	0.73	1.47
**108**	250	2.76	9.93	4.98	28.16	0.42	3.34	13.24	0.50	4.68	0.73	0.54	0.98	-	1.91	1.25	1.49	1.46	2.05	1.82	80.25	0.67	2.56
**108**	400	1.64	2.04	3.45	2.90	0.01	0.44	1.82	0.38	0.41	1.13	0.31	0.06	0.04	0.15	4.01	0.08	0.08	0.47	0.06	19.48	1.58	2.39
**108**	1000	1.21	2.09	2.36	1.98	0.18	0.40	1.54	0.32	0.04	0.58	0.35	0.03	-	0.35	-	-	0.22	0.26	0.04	11.95	2.91	2.19
**108**	1800	1.28	1.36	2.49	1.31	0.37	0.56	1.10	0.05	0.31	0.42	0.20	0.04	-	0.16	0.04	0.20	-	0.19	0.15	10.23	1.92	1.86
**108**	2750	1.27	1.43	2.20	2.04	0.45	0.74	1.39	0.08	0.24	0.53	-	0.04	0.01	0.27	-	-	0.25	0.35	0.25	11.54	-	2.99
**108**	4200	1.32	2.08	1.63	4.38	0.35	0.86	2.46	0.63	0.07	1.08	0.08	0.03	0.07	0.45	-	-	0.12	0.46	0.14	16.20	0.49	2.83
**109**	5	1.83	2.35	6.34	3.74	1.07	1.26	14.45	0.64	0.50	1.77	3.96	0.21	0.17	0.52	-	1.13	1.37	0.35	0.59	42.26	9.36	0.82
**109**	50	2.85	5.26	4.56	5.09	0.44	0.92	2.75	0.70	0.27	1.83	0.97	0.14	0.20	0.51	-	0.65	0.88	0.48	0.41	28.91	3.35	1.66
**109**	100	2.64	5.44	4.05	11.63	0.37	1.59	4.03	2.14	2.17	3.52	0.73	-	0.48	0.33	0.55	0.59	0.47	0.84	0.98	42.57	1.72	1.97
**109**	250	1.20	2.70	3.47	9.96	0.25	1.36	5.15	1.53	0.12	2.81	0.36	0.22	0.38	0.59	0.44	0.63	0.40	0.77	0.67	33.00	1.08	2.35
**109**	500	0.96	1.70	2.95	5.55	0.70	1.22	3.33	1.25	0.12	1.60	0.61	0.09	0.14	0.43	0.47	0.30	0.12	0.44	0.30	22.29	2.75	1.96
**109**	1000	0.76	0.74	2.05	1.75	0.19	0.28	0.23	0.22	0.33	0.44	0.25	0.03	-	-	0.18	0.13	-	0.21	0.53	8.32	3.06	2.55
**109**	1800	1.43	1.78	2.65	2.60	0.58	0.89	0.79	0.01	0.59	0.52	0.21	0.03	0.05	0.08	-	0.09	0.14	0.30	0.01	12.75	1.66	2.39
**109**	2750	1.81	1.40	3.27	2.15	0.60	0.45	1.00	0.26	0.03	0.64	1.23	0.01	-	-	0.18	0.13	-	-	0.53	13.70	9.01	-
**109**	5000	1.00	1.87	1.80	6.03	0.47	1.30	2.13	0.69	1.28	1.68	0.14	0.03	0.20	0.51	0.25	0.23	0.22	0.51	0.30	20.61	0.66	2.46
**111**	8	2.76	4.11	3.04	7.22	0.75	0.43	2.49	0.85	0.32	1.88	1.91	0.12	0.21	0.45	-	0.10	0.15	0.37	0.77	27.95	6.84	1.33
**111**	50	2.12	4.34	7.04	5.25	0.88	0.85	3.50	1.02	0.41	2.66	1.96	0.10	0.22	0.49	0.14	0.28	0.22	0.51	0.41	32.40	6.05	1.57
**111**	100	1.57	2.24	3.82	2.37	0.31	0.16	1.51	0.20	0.03	0.68	0.90	0.04	0.07	0.02	0.01	0.02	-	0.35	0.17	14.47	6.22	2.42
**111**	250	1.93	2.14	3.64	2.42	0.54	0.53	1.25	0.29	0.86	0.54	0.30	0.03	0.02	0.03	0.36	0.36	0.02	0.18	0.51	15.95	1.91	1.15
**111**	500	1.49	1.71	2.88	2.82	0.69	0.81	1.48	0.38	-	0.53	2.44	-	0.02	-	0.25	0.28	-	0.54	1.26	17.60	13.86	3.07
**111**	1000	1.29	1.58	2.14	2.88	0.64	0.86	1.33	0.67	-	0.67	0.31	-	0.04	0.52	0.33	0.65	0.00	0.34	0.02	14.28	2.19	2.38
**111**	1800	1.33	1.83	2.24	2.75	0.60	0.77	1.24	0.37	-	0.59	0.22	-	0.03	0.16	-	-	-	0.35	0.04	12.51	1.76	2.76
**111**	2750	2.51	4.19	1.74	8.83	0.17	0.96	5.00	1.73	0.03	2.33	0.21	0.03	0.53	0.47	0.08	0.43	0.42	0.76	0.60	31.04	0.69	2.45
**111**	4000	2.28	4.88	1.42	11.92	0.17	1.31	6.23	0.52	0.96	3.21	0.22	0.01	0.69	0.77	0.18	0.56	0.61	1.08	0.87	37.91	0.59	2.84
**111**	4830	1.07	1.74	1.54	5.39	0.42	1.01	2.84	0.75	0.02	1.46	0.17	0.02	0.23	0.48	0.27	0.49	0.25	0.56	1.24	19.95	0.86	2.83

Station	Depth	ASP	GLU	ASN	SER	GLN	HIS	GLY	ARG	THR	ALA	TAU	GABA	TYR	VAL/MET	TRP	PHE	ILE	LEU	LYS	total DFAA	Tau/DFAA [%]	Leu/DFAA [%]
**56**	6	1.55	2.50	7.87	5.10	0.81	0.80	4.20	1.26	0.97	1.91	2.10	0.14	0.30	0.72	0.04	0.22	0.21	0.53	0.60	31.82	6.60	1.66
**56**	60	1.76	4.11	4.21	5.59	2.19	3.28	3.95	0.59	0.76	3.20	9.38	0.20	0.36	0.45	0.12	0.16	0.17	0.47	0.58	41.52	22.59	1.14
**56**	90	1.22	1.89	5.36	2.90	0.19	0.58	1.38	0.37	10.34	0.83	0.81	0.06	0.08	-	-	0.01	2.20	0.27	0.13	28.61	2.82	0.93
**56**	100	1.58	2.75	4.44	4.10	0.93	1.14	0.53	1.49	0.74	1.08	0.36	0.06	0.04	0.09	-	0.16	-	0.28	0.15	19.89	1.79	1.39
**56**	250	1.32	2.06	3.57	2.94	0.51	-	1.66	0.41	0.03	0.92	0.42	0.03	0.06	0.18	-	0.36	0.04	0.21	0.13	14.84	2.80	1.42
**56**	500	1.53	2.78	2.12	5.11	0.63	1.12	2.10	0.24	0.89	1.34	0.45	0.04	0.17	0.12	-	0.11	0.08	0.35	0.26	19.42	2.33	1.80
**56**	1000	0.91	0.86	2.42	1.55	0.10	0.31	0.26	0.41	0.36	0.42	0.43	0.05	0.02	-	-	-	-	0.39	0.01	8.50	5.08	4.59
**56**	2070	0.82	1.00	1.92	3.61	0.48	0.87	2.57	0.52	0.25	1.03	0.40	-	0.40	0.08	-	-	0.06	0.35	0.20	14.57	2.77	2.40
**56**	2750	0.94	1.57	1.41	4.13	0.34	0.68	2.21	0.48	0.25	0.94	0.26	0.06	0.17	0.24	0.25	0.43	0.07	0.43	0.18	15.07	1.75	2.87
**57**	6	1.39	1.26	6.40	1.61	1.43	0.66	0.07	-	1.29	0.59	4.23	0.07	-	0.08	0.31	0.24	-	0.12	0.14	19.89	21.24	0.61
**57**	50	2.19	3.04	5.56	1.63	0.08	0.75	0.71	0.22	0.38	0.87	1.50	-	-	0.14	-	-	0.64	0.19	0.24	18.14	8.29	1.07
**57**	100	1.22	1.69	3.95	2.47	0.52	0.76	0.94	0.36	0.07	0.69	0.92	-	-	0.43	0.22	0.15	-	0.19	0.16	14.74	6.26	1.31
**57**	250	0.89	0.80	2.81	1.66	0.63	0.46	0.28	0.03	0.01	0.27	0.64	0.03	-	0.23	0.14	0.13	-	0.18	0.07	9.26	6.93	1.92
**57**	500	0.89	0.80	2.40	0.70	0.29	0.21	0.20	-	-	0.21	0.63	-	-	0.26	-	-	-	0.12	0.19	6.88	9.09	1.68
**57**	1000	0.68	0.63	0.68	0.28	0.32	0.19	-	-	-	-	0.27	-	-	0.02	-	-	-	0.13	-	3.19	8.40	4.02
**57**	1900	0.95	0.73	1.02	-	-	-	-	-	-	-	0.28	0.03	-	-	-	-	-	0.03	-	3.04	9.24	0.93
**57**	2750	0.90	1.22	1.24	3.71	-	0.55	1.87	0.56	0.11	1.19	0.42	-	-	1.41	0.23	0.09	-	0.41	0.18	14.07	2.95	2.90
**57**	3150	0.97	0.78	1.42	1.07	0.17	0.25	0.13	0.03	-	0.12	0.28	0.03	-	0.03	-	0.03	0.21	0.19	0.19	5.90	4.69	3.23
**58**	5	1.73	1.67	7.36	-	-	-	0.04	-	0.46	0.61	1.10	-	-	0.05	0.05	-	0.17	0.36	-	13.61	8.06	2.63
**58**	60	1.38	1.63	5.56	1.04	-	0.95	0.39	-	0.27	0.55	1.19	0.03	-	0.13	-	0.53	-	0.14	-	13.78	8.66	1.01
**58**	100	1.20	6.26	3.68	20.13	-	-	10.55	3.97	-	5.49	1.57	-	-	1.26	1.08	1.74	1.18	1.88	2.23	62.23	2.52	3.03
**58**	250	0.94	0.49	3.72	0.56	-	-	-	-	-	-	0.80	0.04	-	0.09	-	0.82	-	-	-	7.46	10.69	-
**58**	500	0.78	1.51	2.67	6.20	-	1.61	3.03	2.01	0.48	2.00	0.61	0.03	-	0.49	-	0.14	0.08	0.54	0.43	22.62	2.69	2.38
**58**	1000	0.68	0.39	1.86	0.50	-	0.31	-	-	0.00	0.01	0.59	-	-	-	-	-	0.10	0.13	-	4.57	12.86	2.79
**58**	1800	1.18	1.41	1.70	1.06	-	0.46	0.49	-	0.14	0.24	0.39	-	-	0.02	-	-	-	0.17	0.11	7.38	5.29	2.37
**59**	5	1.46	1.49	8.03	1.39	2.04	0.49	1.12	0.51	1.17	2.08	3.36	-	-	0.10	-	-	-	0.15	0.15	23.53	14.26	0.63
**59**	70	2.60	2.26	5.79	7.60	0.47	1.79	4.46	1.49	0.34	2.78	1.89	-	-	0.38	0.26	0.23	0.19	0.38	1.17	34.07	5.55	1.11
**59**	100	0.92	0.72	4.34	0.58	0.73	0.73	0.17	-	0.11	0.22	1.16	-	-	-	0.87	-	-	0.13	-	10.67	10.86	1.23
**59**	250	0.74	0.51	3.09	-	-	0.34	-	-	0.29	-	0.39	-	-	-	-	0.02	-	0.10	-	5.47	7.13	1.77
**59**	500	0.89	0.91	4.41	-	-	0.72	0.27	-	0.24	0.12	0.36	-	-	0.23	0.11	0.05	-	0.14	-	8.45	4.21	1.66
**59**	100	1.05	0.95	3.88	0.04	-	0.59	0.65	0.21	-	0.65	0.23	-	-	0.32	-	0.61	-	0.20	0.18	9.56	2.41	2.07
**59**	1900	0.98	1.15	3.12	1.59	0.40	0.67	0.85	0.22	0.01	0.67	0.20	0.04	-	0.11	0.12	0.08	-	0.30	0.25	10.75	1.83	2.78
**59**	2700	0.94	0.86	1.12	1.33	0.15	0.41	0.47	0.17	0.04	0.37	0.35	-	-	0.16	0.09	0.03	-	0.20	0.06	6.74	5.23	2.94

Station	Depth	ASP	GLU	ASN	SER	GLN	HIS	GLY	ARG	THR	ALA	TAU	GABA	TYR	VAL/MET	TRP	PHE	ILE	LEU	LYS	total DFAA	Tau/DFAA [%]	Leu/DFAA [%]
**60**	5	1.90	2.52	7.83	3.18	1.36	0.75	3.20	0.73	0.43	1.39	2.91	-	0.10	0.44	0.38	0.42	0.23	0.47	0.53	28.77	10.12	1.63
**60**	60	1.22	1.50	5.71	2.65	0.76	0.75	0.85	0.29	0.14	0.91	0.86	-	-	0.17	-	0.38	0.06	0.23	0.26	16.73	5.15	1.36
**60**	100	1.11	1.38	9.19	0.44	0.82	1.36	2.21	0.85	0.37	1.45	0.95	0.12	-	0.18	-	0.28	0.04	0.31	0.20	21.26	4.48	1.47
**60**	250	0.86	1.82	8.69	-	-	0.81	2.38	0.24	0.31	1.53	0.79	0.05	-	0.18	-	0.78	0.07	0.28	0.37	19.16	4.14	1.49
**60**	500	0.87	0.86	2.70	0.59	-	0.49	-	0.84	0.18	-	-	0.03	-	0.28	0.30	0.10	-	0.17	-	7.42	-	2.31
**60**	1000	1.40	1.99	2.55	2.42	0.63	0.77	0.77	0.03	0.30	0.64	1.08	-	-	4.66	-	0.94	0.14	0.28	0.17	18.77	5.75	1.50
**60**	1900	0.83	0.93	3.49	1.06	0.48	0.68	0.78	0.38	0.41	0.60	0.18	0.01	-	1.07	0.39	-	0.37	0.32	0.32	12.32	1.50	2.59
**60**	2450	1.37	2.35	4.37	1.85	0.43	0.88	1.84	0.89	0.09	1.22	0.19	0.03	-	0.21	-	-	0.08	0.38	0.58	16.77	1.12	2.26
**62**	5	1.54	1.93	8.68	1.70	2.67	1.28	8.63	0.43	1.08	2.11	4.94	0.09	-	0.15	0.16	0.09	0.06	0.14	0.68	36.37	13.57	0.39
**62**	30	1.41	2.36	6.66	1.28	1.91	0.31	2.72	0.11	0.48	0.52	5.88	-	-	0.18	-	-	0.24	0.19	0.33	24.60	23.92	0.75
**62**	50	1.18	1.53	5.08	0.83	1.51	0.55	1.29	-	0.33	0.84	3.70	0.03	-	0.06	0.02	0.04	-	0.14	-	17.13	21.63	0.80
**62**	75	1.56	1.55	4.06	0.65	0.07	0.28	0.30	-	0.01	0.32	0.76	-	-	0.06	-	0.10	-	0.13	-	9.84	7.68	1.32
**62**	220	0.79	0.55	3.60	0.55	0.63	0.58	0.06	-	0.06	0.14	1.87	-	-	-	-	0.12	-	0.17	-	9.13	20.52	1.84
**64**	5	1.67	4.06	8.12	3.23	1.50	1.80	9.34	0.59	0.70	1.97	5.55	-	0.02	0.47	0.49	0.91	0.08	0.48	0.51	41.51	13.38	1.15
**64**	10	1.30	3.76	14.83	0.51	0.84	2.02	14.41	1.62	1.09	4.87	6.88	0.12	0.10	0.59	0.17	0.44	0.18	0.52	0.73	54.98	12.51	0.95
**64**	20	1.79	1.56	4.69	2.18	0.58	0.49	3.03	0.31	0.20	0.27	1.33	0.07	-	0.13	0.04	0.03	-	0.18	0.06	16.94	7.86	1.08
**64**	50	2.30	6.20	19.48	1.99	0.71	2.68	7.81	2.35	1.30	5.28	1.37	0.09	0.37	1.08	0.63	0.71	0.67	0.94	2.77	58.72	2.33	1.60
**114**	4	2.86	3.13	6.69	3.63	0.81	0.66	2.21	1.04	0.90	1.14	1.66	0.03	0.22	0.25	-	0.14	0.13	0.38	0.35	26.24	6.34	1.46
**114**	70	1.60	3.43	5.19	3.23	0.75	0.30	2.20	0.36	0.51	1.59	2.51	-	0.10	0.33	-	0.16	0.05	0.25	-	22.56	11.13	1.09
**114**	100	1.42	2.42	4.06	2.64	0.97	1.19	2.19	0.51	0.22	0.98	0.92	-	0.06	0.10	-	0.06	0.56	0.30	0.11	18.71	4.92	1.58
**114**	250	0.96	1.24	5.09	2.11	0.70	0.89	1.56	0.25	0.15	0.58	0.45	0.06	0.08	-	-	-	0.19	0.24	0.06	14.61	3.09	1.66
**114**	500	0.72	0.51	4.06	1.73	0.70	0.75	1.33	0.16	0.01	0.47	0.38	0.05	-	0.17	-	0.01	-	0.18	0.02	11.25	3.34	1.63
**114**	1000	1.10	1.52	2.13	1.59	0.46	0.69	1.20	0.00	0.13	0.51	0.25	0.08	-	0.05	-	-	0.73	0.25	-	10.70	2.32	2.34
**114**	1760	1.76	1.31	2.66	2.01	0.48	0.34	1.16	0.29	-	0.35	0.21	0.03	-	-	-	0.03	-	0.17	0.18	10.99	1.89	1.54
**114**	2750	1.40	1.98	1.95	3.70	0.49	0.88	2.20	0.51	0.12	0.84	0.18	0.09	0.09	0.20	-	0.27	0.08	0.39	0.13	15.51	1.17	2.52
**114**	3700	0.97	2.07	2.11	3.98	0.27	1.63	2.63	1.57	0.10	1.88	0.28	0.12	0.40	0.51	-	0.73	0.33	0.66	0.58	20.81	1.36	3.16
**115**	10	1.98	2.09	6.14	5.07	1.43	1.23	2.69	0.54	0.66	1.40	0.84	0.06	0.15	0.21	-	-	1.02	0.49	0.54	26.55	3.15	1.84
**115**	50	1.95	2.70	5.92	2.34	0.99	1.11	2.18	0.21	0.09	1.28	1.24	0.20	0.13	0.36	0.50	0.42	1.31	0.23	0.82	23.99	5.17	0.94
**115**	100	1.49	1.50	5.01	2.20	0.05	0.30	1.44	0.13	0.34	0.70	1.03	0.04	0.01	0.02	-	-	-	0.40	0.02	14.69	7.00	2.74
**115**	250	1.46	1.67	4.51	2.56	0.72	0.92	1.48	-	0.34	0.28	0.44	0.27	0.07	0.05	-	-	0.29	0.31	0.26	15.64	2.81	1.98
**115**	500	1.06	1.45	4.90	2.58	0.57	1.01	2.11	0.51	0.16	1.28	0.55	0.03	0.09	0.17	-	-	0.19	0.42	0.14	17.21	3.18	2.47
**115**	1000	1.53	1.68	2.69	2.06	0.01	0.30	1.37	0.23	-	0.54	0.39	0.05	-	0.12	-	0.15	-	0.21	-	11.32	3.47	1.85
**115**	2000	1.36	2.18	3.93	0.88	0.39	0.66	1.60	0.33	0.05	0.78	0.18	0.05	0.02	0.01	0.09	0.03	-	0.37	0.33	13.23	1.32	2.79
**115**	2750	1.31	1.22	1.93	1.95	0.53	0.61	-	0.75	0.38	0.31	0.25	-	-	0.21	0.13	0.49	-	0.31	-	10.36	2.43	2.99
**115**	3950	1.32	1.35	1.50	4.23	0.22	0.31	2.29	0.89	0.10	1.17	0.20	0.03	0.13	0.31	-	0.05	0.10	0.31	0.24	12.47	1.57	2.49

Table S4b. Dissolved free amino acids (DFAA) throughout the water column at the stations occupied during the RadProf cruise in the North Atlantic Ocean. The total DFAA and the percentages of dissolved taurine and leucine to the total DFAA [%] are also indicated. Concentrations between the detection and quantification limits are marked grey. Abbreviations: Asp, Aspartic acid; Glu, Glutamic acid; Asn, Asparagine; Ser, Serine; Gln, Glutamine; His, Histine; Gly, Glycine; Arg, Arginine; Thr, Threonine; Ala, Alanine; Tau, Taurine; GABA, gamma aminobutyric acid; Tyr, Tyrosine; Met, Methionine; Val, Valine; Trp, Tryptophan; Phe, Phenylalanine; Ile, Isoleucine; Leu, Leucine; Lys, Lysine.

Station	Depth	ASP	GLU	ASN	SER	GLN	HIS	GLY	ARG	THR	ALA	TAU	GABA	TYR	VAL/MET	TRP	PHE	ILE	LEU	LYS	total DFAA	Tau/DFAA [%]	Leu/DFAA [%]
**8**	5	2.48	2.82	11.14	0.52	0.51	0.35	2.62	0.08	0.57	2.75	5.72	0.10	0.07	0.59	-	0.25	0.40	1.29	0.25	32.51	17.58	3.98
**8**	50	3.40	3.85	10.66	-	0.86	0.77	3.07	0.28	0.49	2.58	5.96	0.29	-	0.50	1.08	0.51	0.03	1.67	0.39	36.37	16.38	4.59
**8**	100	2.53	15.13	7.70	1.60	0.21	0.10	1.42	0.45	-	0.40	0.83	0.28	0.20	0.50	-	-	0.01	0.56	0.25	32.16	2.58	1.73
**8**	250	1.85	2.43	8.15	2.03	-	-	0.12	-	-	0.07	0.93	0.21	0.14	0.14	-	-	-	0.05	-	16.12	5.79	0.30
**8**	500	1.59	1.15	7.78	-	0.17	-	0.91	0.14	-	0.20	0.73	0.20	0.11	-	-	-	-	0.23	0.39	13.60	5.36	1.71
**8**	1000	1.27	1.47	6.27	0.79	0.44	-	0.06	-	0.06	0.03	0.77	0.28	0.12	-	-	0.91	0.50	0.01	-	12.97	5.95	0.09
**8**	1900	1.38	1.34	5.74	1.42	0.43	0.05	0.38	0.10	0.09	0.26	1.36	0.17	0.07	-	-	0.22	0.36	0.58	-	13.96	9.77	4.17
**11**	5	0.79	2.34	10.49	4.04	0.58	0.25	1.39	0.73	0.07	1.09	1.35	0.03	0.09	0.31	0.98	0.36	0.41	1.19	0.12	26.60	5.08	4.46
**11**	100	1.40	2.40	13.14	1.40	0.49	0.14	-	3.70	0.88	1.16	0.62	0.39	0.21	0.56	1.84	0.39	0.17	0.46	0.55	29.91	2.06	1.52
**11**	250	1.73	2.25	9.24	-	-	-	0.98	0.19	0.06	0.35	0.63	0.25	0.12	0.35	0.66	0.20	0.06	0.29	0.25	17.59	3.59	1.63
**11**	400	2.87	1.88	8.00	0.06	-	-	0.81	0.05	0.09	-	0.45	0.03	0.04	0.33	0.35	0.23	-	0.23	0.16	15.56	2.87	1.45
**11**	700	3.78	3.66	6.06	2.07	2.08	0.80	-	-	-	0.50	0.74	0.10	0.11	0.50	1.28	0.66	0.06	0.29	0.53	23.22	3.19	1.26
**11**	1000	1.68	1.60	6.48	0.43	1.61	-	-	3.85	0.53	1.03	0.52	0.27	0.24	0.42	1.42	0.36	0.06	0.43	0.52	21.42	2.41	1.99
**11**	1800	2.13	1.45	6.29	1.42	0.12	0.30	1.58	0.65	0.10	0.83	0.29	0.26	0.12	0.60	1.34	0.34	0.08	0.47	0.34	18.72	1.55	2.53
**11**	2500	2.03	1.25	7.45	-	-	-	0.85	0.09	-	0.07	0.25	-	0.01	0.53	1.36	0.97	0.03	0.22	0.50	15.62	1.58	1.41
**16**	5	1.12	2.01	4.00	2.61	0.21	0.36	1.35	0.80	0.27	0.72	1.20	0.19	0.06	-	0.42	0.80	0.38	1.40	0.41	18.31	6.55	7.65
**16**	38	1.31	2.29	10.11	6.48	1.68	0.60	-	1.16	0.08	-	0.85	-	-	0.33	0.03	-	0.41	-	-	25.33	3.34	-
**16**	100	2.03	2.92	11.29	-	0.19	0.45	1.40	0.20	0.07	0.46	1.14	0.20	0.18	0.28	0.89	0.40	0.04	0.41	0.20	22.74	5.03	1.79
**16**	350	2.11	2.61	7.92	1.63	0.53	0.21	1.06	0.23	0.09	0.42	1.01	0.14	0.15	0.36	-	-	-	0.49	0.21	19.14	5.26	2.54
**16**	600	1.18	1.70	6.40	1.32	0.21	-	0.71	0.13	0.10	0.13	0.28	0.15	0.12	0.33	0.42	0.18	-	0.33	0.09	13.79	2.01	2.38
**16**	1000	2.30	2.12	5.79	2.03	0.12	0.25	1.34	0.57	0.13	0.78	0.42	0.22	0.12	0.48	-	-	0.13	0.41	0.29	17.49	2.37	2.36
**16**	2000	0.77	0.78	3.99	-	-	-	-	-	-	0.94	0.96	-	0.01	0.20	-	0.14	0.27	2.04	2.80	12.90	7.44	15.84
**16**	2450	1.92	1.52	5.22	1.83	0.13	0.21	1.14	0.35	0.09	0.55	0.28	0.25	0.24	0.21	0.55	0.33	-	0.61	0.25	15.69	1.80	3.91

Station	Depth	ASP	GLU	ASN	SER	GLN	HIS	GLY	ARG	THR	ALA	TAU	GABA	TYR	VAL/MET	TRP	PHE	ILE	LEU	LYS	total DFAA	Tau/DFAA [%]	Leu/DFAA [%]
**108**	5	1.03	1.25	4.97	9.77	0.17	0.96	6.07	3.03	0.40	3.50	1.22	1.02	0.28	1.24	3.11	0.26	0.91	1.44	3.86	44.47	2.73	3.23
**108**	100	1.90	4.81	9.06	5.86	0.29	0.83	2.88	1.73	0.31	2.01	0.92	0.37	0.37	0.42	1.82	0.34	0.30	0.60	0.83	35.63	2.57	1.69
**108**	400	2.26	3.12	7.83	1.21	0.56	0.30	1.12	0.18	0.13	0.43	1.03	0.11	0.17	0.35	1.06	0.41	-	0.26	0.26	20.78	4.94	1.26
**108**	550	1.19	1.88	5.95	1.73	0.19	0.22	1.12	0.42	0.18	0.61	0.59	0.23	0.15	0.20	0.51	0.09	0.10	0.36	0.44	16.15	3.66	2.20
**108**	1000	1.35	1.54	-	3.01	1.08	-	-	1.31	0.26	0.19	0.21	0.16	0.10	0.33	1.01	0.36	0.08	0.30	0.25	11.55	1.80	2.64
**108**	2000	2.02	4.57	5.67	-	-	-	0.81	0.28	0.17	0.14	0.40	0.04	0.01	0.46	0.56	0.10	-	1.41	0.22	16.86	2.37	8.35
**108**	2750	1.92	1.85	4.29	1.47	0.08	0.06	1.09	0.41	0.03	0.74	-	0.13	0.07	0.24	0.40	0.09	0.12	0.56	0.54	14.08	-	3.97
**108**	4000	3.09	2.69	4.10	1.53	0.17	0.19	0.89	0.35	0.06	0.34	0.09	0.16	0.08	0.21	0.62	0.15	0.06	0.43	0.25	15.45	0.58	2.79
**111**	5	3.81	11.00	52.78	2.59	0.98	3.07	11.31	2.12	2.33	9.91	3.16	0.46	2.22	0.79	0.74	1.49	0.64	5.60	0.17	115.18	2.74	4.86
**111**	100	7.49	5.90	24.09	0.30	1.46	1.99	2.17	3.32	2.27	2.74	0.47	1.83	2.46	0.68	2.12	1.17	0.77	0.94	0.97	63.16	0.74	1.48
**111**	300	7.49	5.90	9.81	0.80	0.10	0.34	2.25	0.74	0.09	1.31	0.53	0.10	0.12	0.41	1.09	0.24	0.04	2.35	0.41	34.10	1.56	6.89
**111**	1000	1.79	3.77	7.48	-	0.33	-	0.41	0.01	0.02	0.03	0.63	-	0.06	0.26	0.50	0.19	-	0.14	0.10	15.71	4.04	0.86
**111**	2000	1.74	2.30	6.34	-	-	0.35	0.57	0.05	0.03	0.11	0.19	0.07	-	0.29	0.73	0.54	0.03	0.44	0.15	13.93	1.38	3.19
**111**	2750	1.36	4.03	3.98	0.78	0.26	-	0.23	0.10	-	0.95	1.63	0.25	0.09	0.29	1.40	0.02	0.19	0.93	0.25	16.72	9.72	5.56
**111**	3200	1.47	1.99	3.88	0.03	0.76	-	-	-	-	0.17	0.16	0.12	-	0.21	0.66	-	0.01	0.42	-	9.87	1.62	4.28
**111**	4500	1.47	2.17	3.44	0.23	0.16	-	0.14	-	0.02	0.31	0.20	0.03	-	0.41	-	-	0.03	0.12	-	8.73	2.33	1.35

Table S5. Slope, coefficient of determination (R^2) and number of stations (n) from the correlation between depth and taurine assimilation and respiration, leucine incorporation and prokaryotic abundance. Abbreviations: SD, standard deviation. (Transect 1 = MODUPLAN + RADPROF).

	n Transect 1	*Slope Transect 1 (Mean ± SD)*	*R^2 Transect 1 (Mean ± SD)*	*n Transect 2*	*slope Transect 2 (Mean ± SD)*	*R^2 Transect 2 (Mean ± SD)*
Taurine assimilation	11	-0.86 ± 0.12	0.802 ± 0.11	7	-0.96 ± 0.28	0.741 ± 0.07
Taurine respiration	11	-1.23 ± 0.38	0.740 ± 0.22	7	-0.92 ± 0.13	0.742 ± 0.06
Leucine incorporation	14	-0.82 ± 0.22	0.815 ± 0.15	9	-0.85 ± 0.27	0.748 ± 0.18
Prokaryotic abundance	14	-1.82 ± 0.76	0.822 ± 0.12	9	-1.55 ± 0.19	0.869 ± 0.07

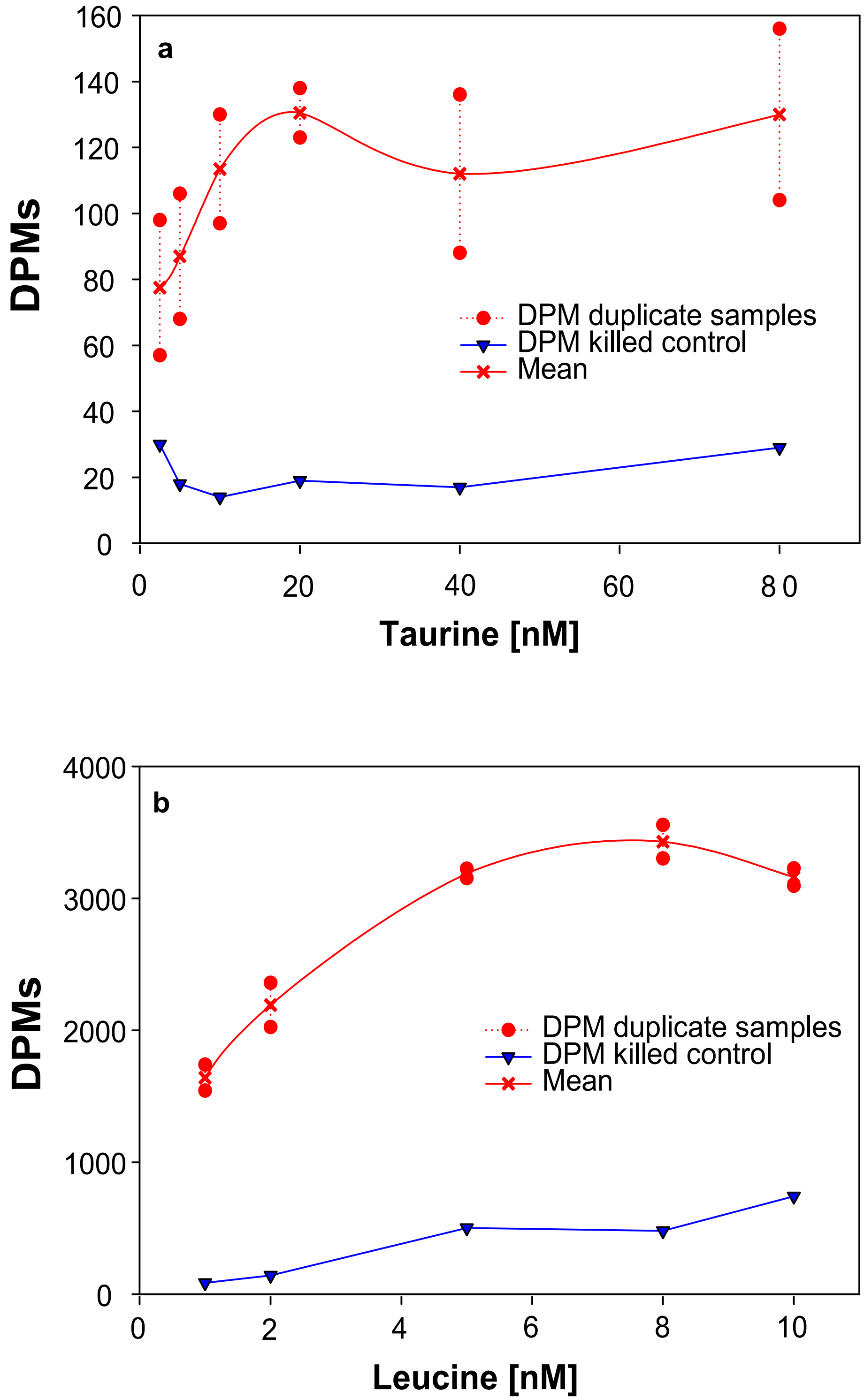

Fig. S1. Saturation curve for (a) taurine and (b) leucine assimilation of prokaryotic communities collected at 2000 m depth. Measured rates correspond to 0.015 nmol taurine $L^{-1}d^{-1}$ and 0.0005 nmol leucine L^{-1} d^{-1}, respectively.

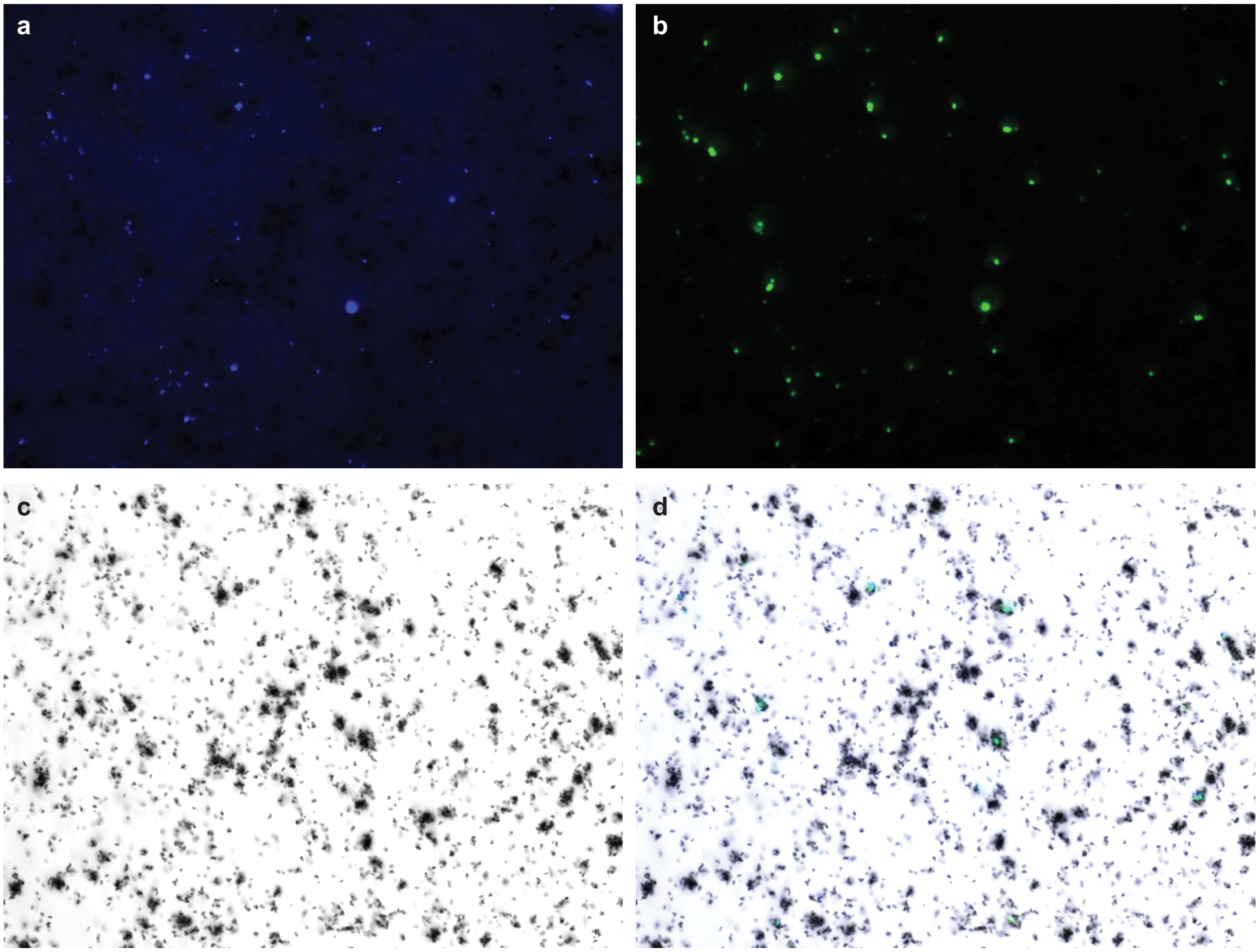

Fig. S2. MICRO-CARD-FISH image of (a) DAPI-stained prokaryotic cells (blue), (b) Bacteria labelled by CARD-FISH (green), (c) silver grains (black), and (d) overlay of the three images. Sample was collected at 5 m depth at Station 8.

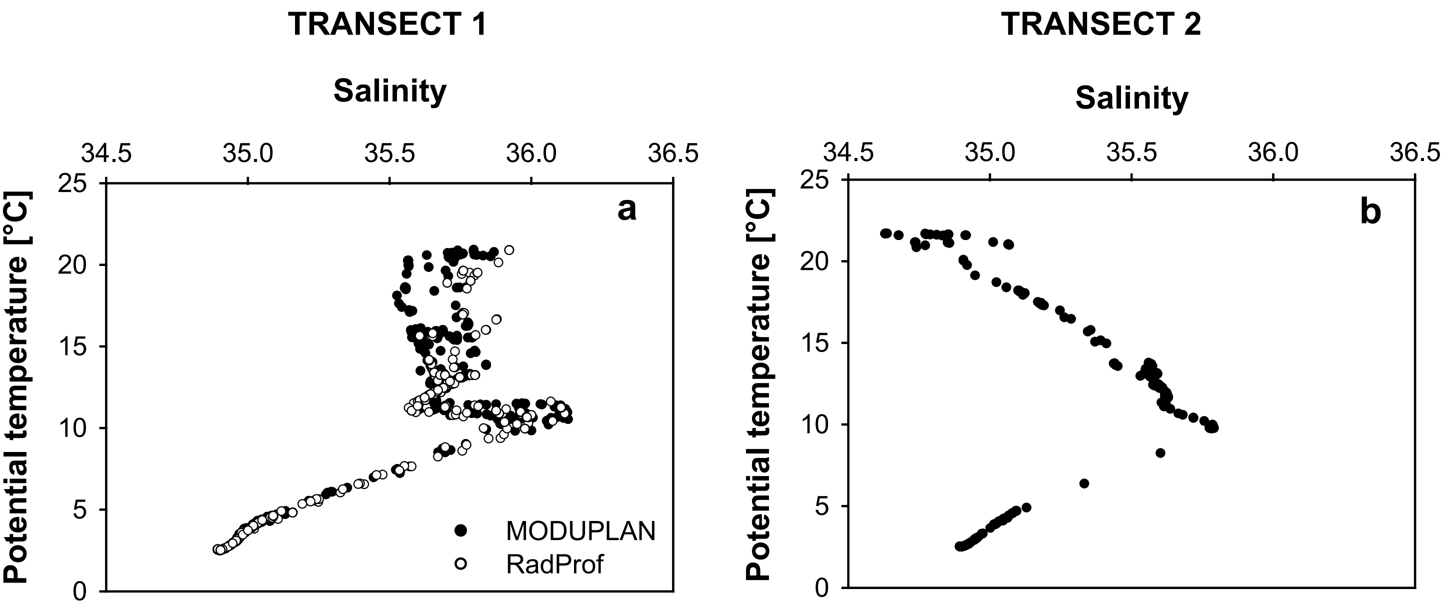

Fig. S3. Temperature-salinity diagrams (Transect 1: a; Transect 2: b) throughout the water column at the stations occupied during the MODUPLAN and the RadProf cruise in the North Atlantic. The data for the MODUPLAN cruise were published in Clifford et al. [7].

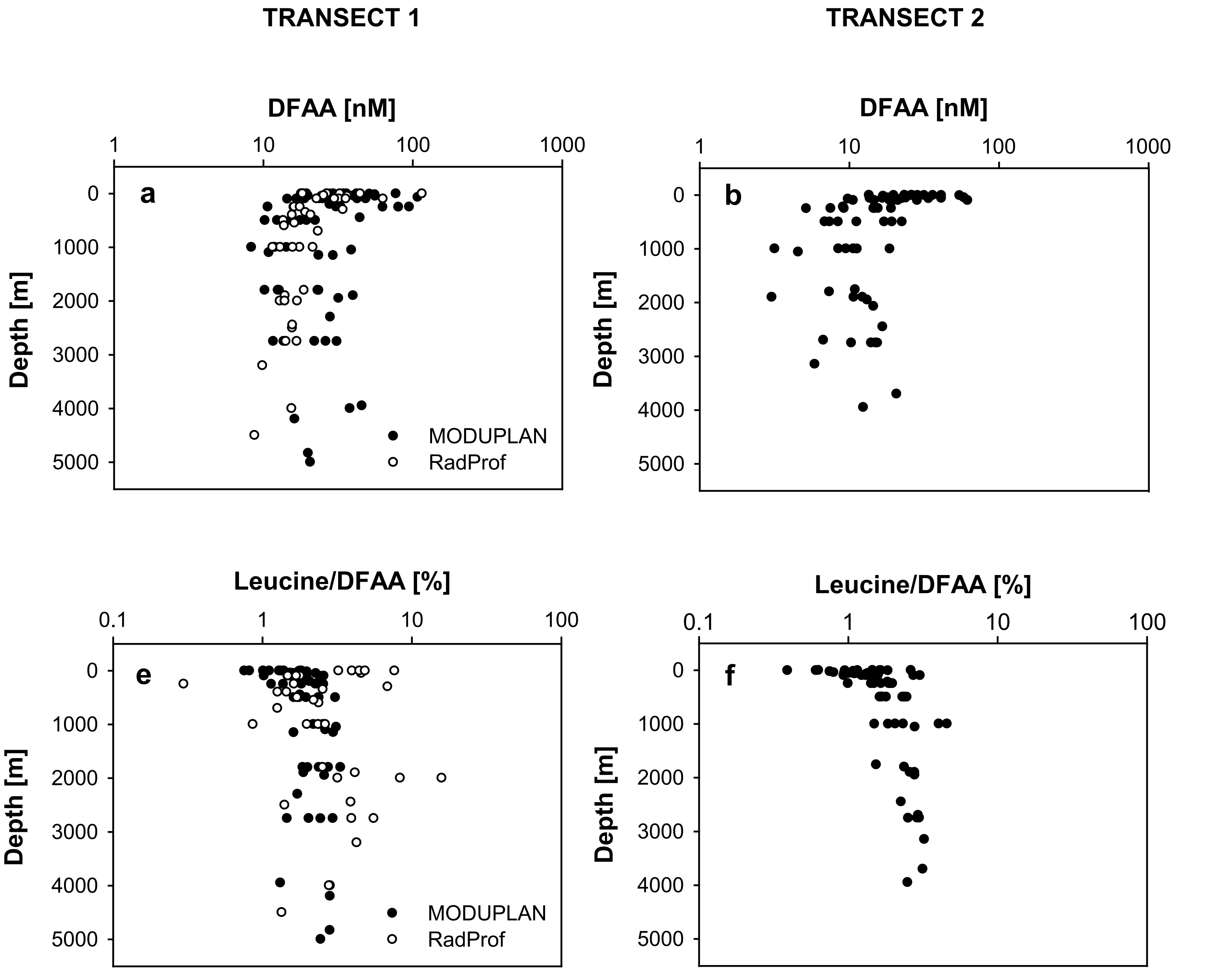

Fig. S4. Dissolved free amino acids (DFAA) concentrations (a, b), and Leucine/DFAA % (c, d) throughout the water column at the stations occupied during the MODUPLAN and the RadProf cruise in the North Atlantic. Data of Transect 1 are shown in the left panels, and data of Transect 2 are shown in the right panels.

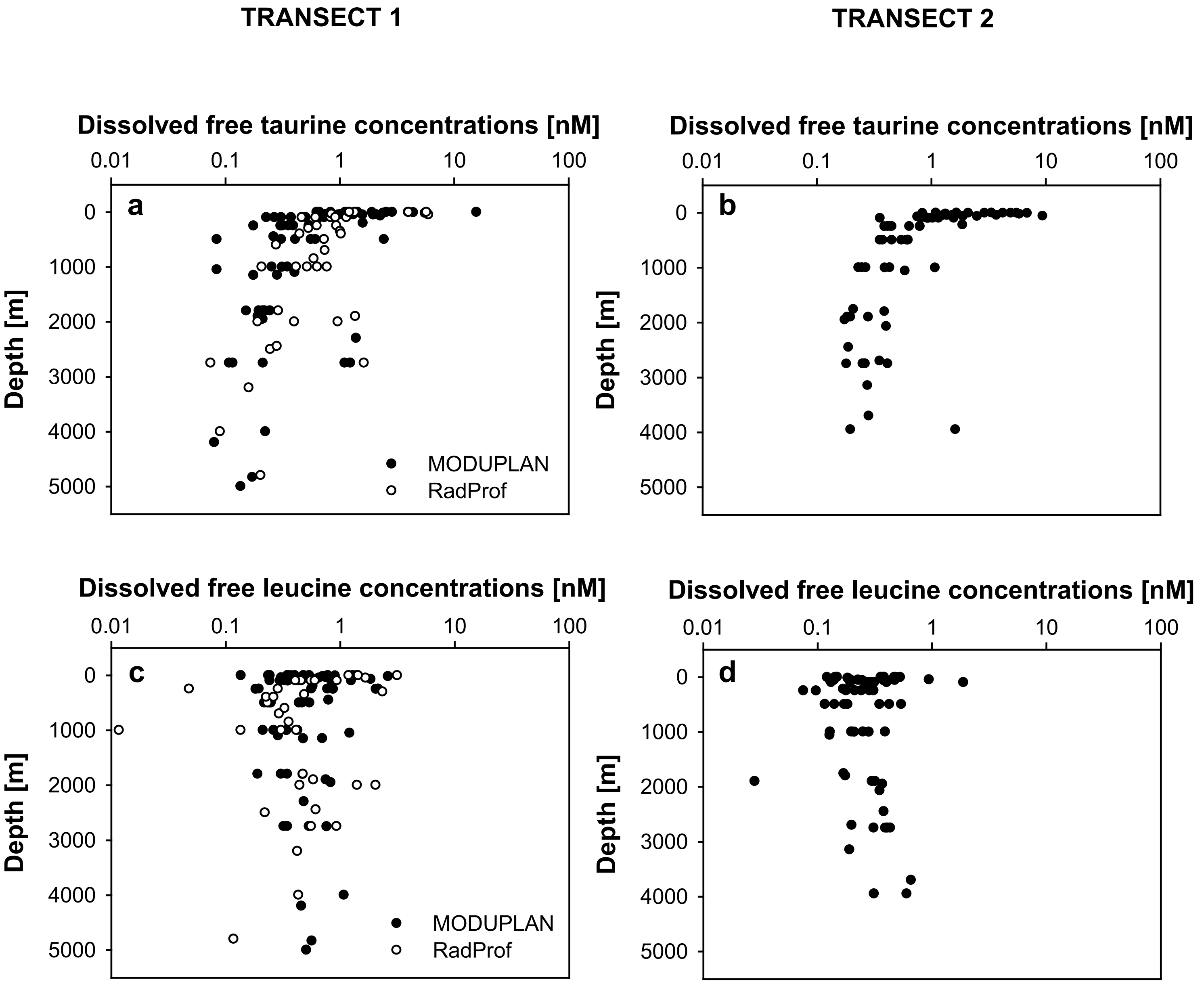

Fig. S5. Dissolved free taurine (a, b) and dissolved free leucine (c, d) concentrations throughout the water column at the stations occupied during the MODUPLAN and the RadProf cruise in the North Atlantic. Left panels show data for Transect 1 and the right panels for Transect 2. The taurine concentrations of the MODUPLAN cruise are from Clifford et al. [7].

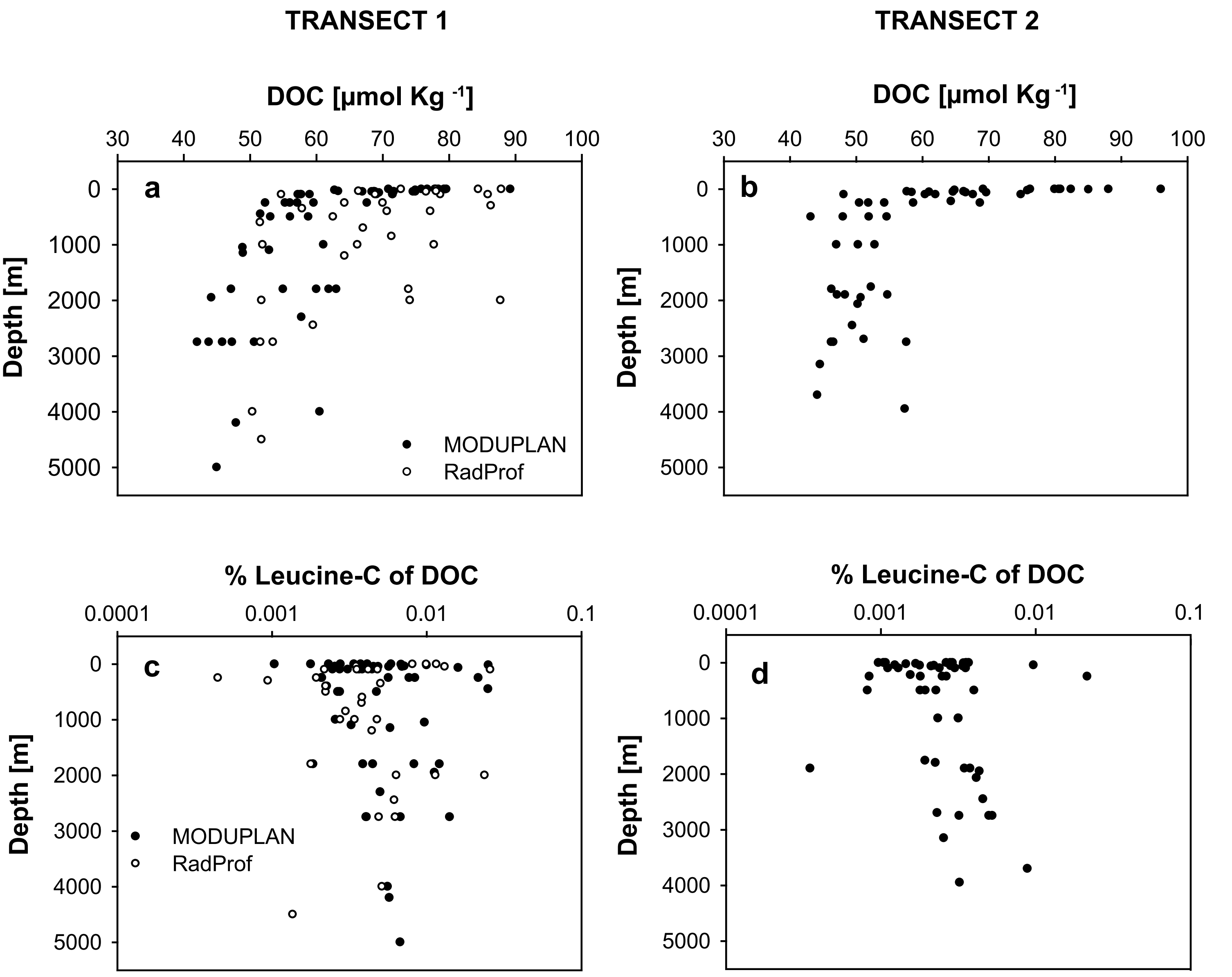

Fig. S6. Dissolved organic matter (DOC) concentrations (a, b), % Leucine-C of DOC (c, d) throughout the water column at the stations occupied during the MODUPLAN and the RadProf cruise in the North Atlantic. Data of Transect 1 are shown in the left panels and data of Transect 2 in the right panels.

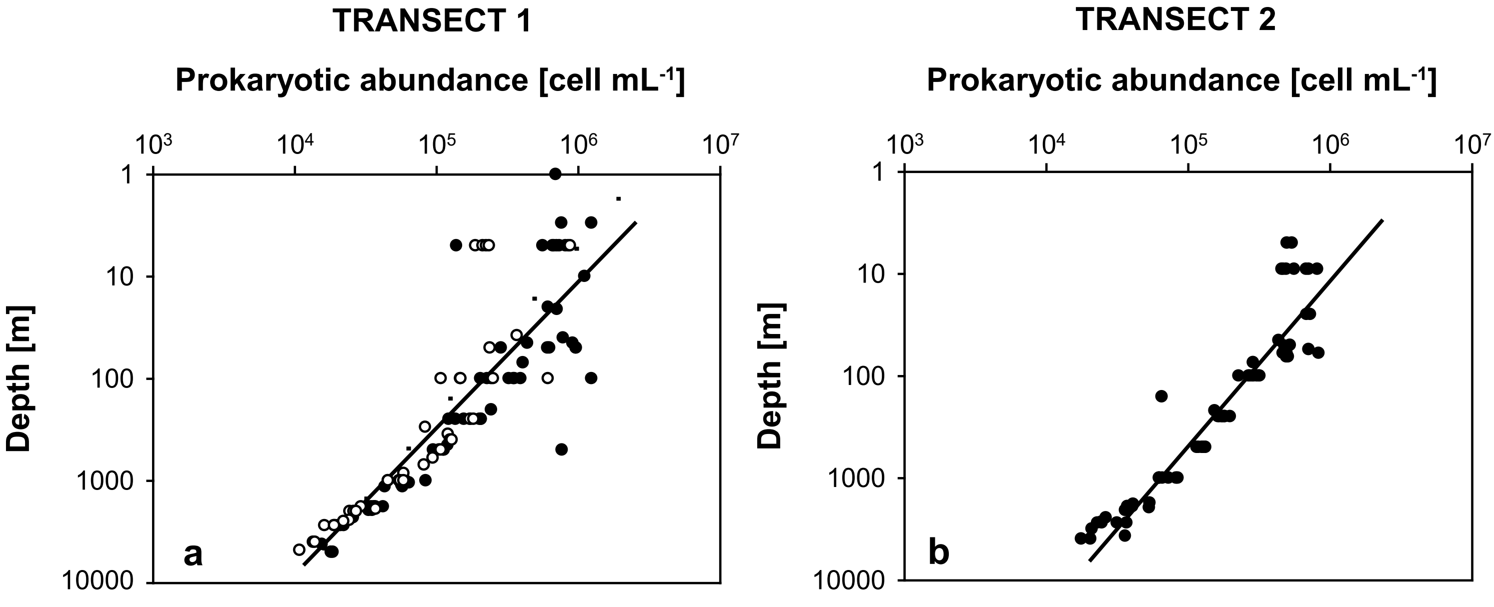

Fig. S7. Log - log plots of prokaryotic abundance determined via flow cytometry (FCM) throughout the water column of (a) Transect 1 (MODUPLAN - epipelagic: n = 31; mesopelagic: n = 18, bathypelagic: n = 21; RadProf - epipelagic: n = 12; mesopelagic: n = 15, bathypelagic: n = 12) and (b) Transect 2 (MODUPLAN - epipelagic: n = 30; mesopelagic: n = 21, bathypelagic: n = 17) at the stations occupied during the MODUPLAN and the RadProf Cruise in the North Atlantic Ocean. The full lines represent the trend-lines of the prokaryotic abundance with depth. The mean slopes and the mean coefficients of determination (R^2) are given in Table S5.

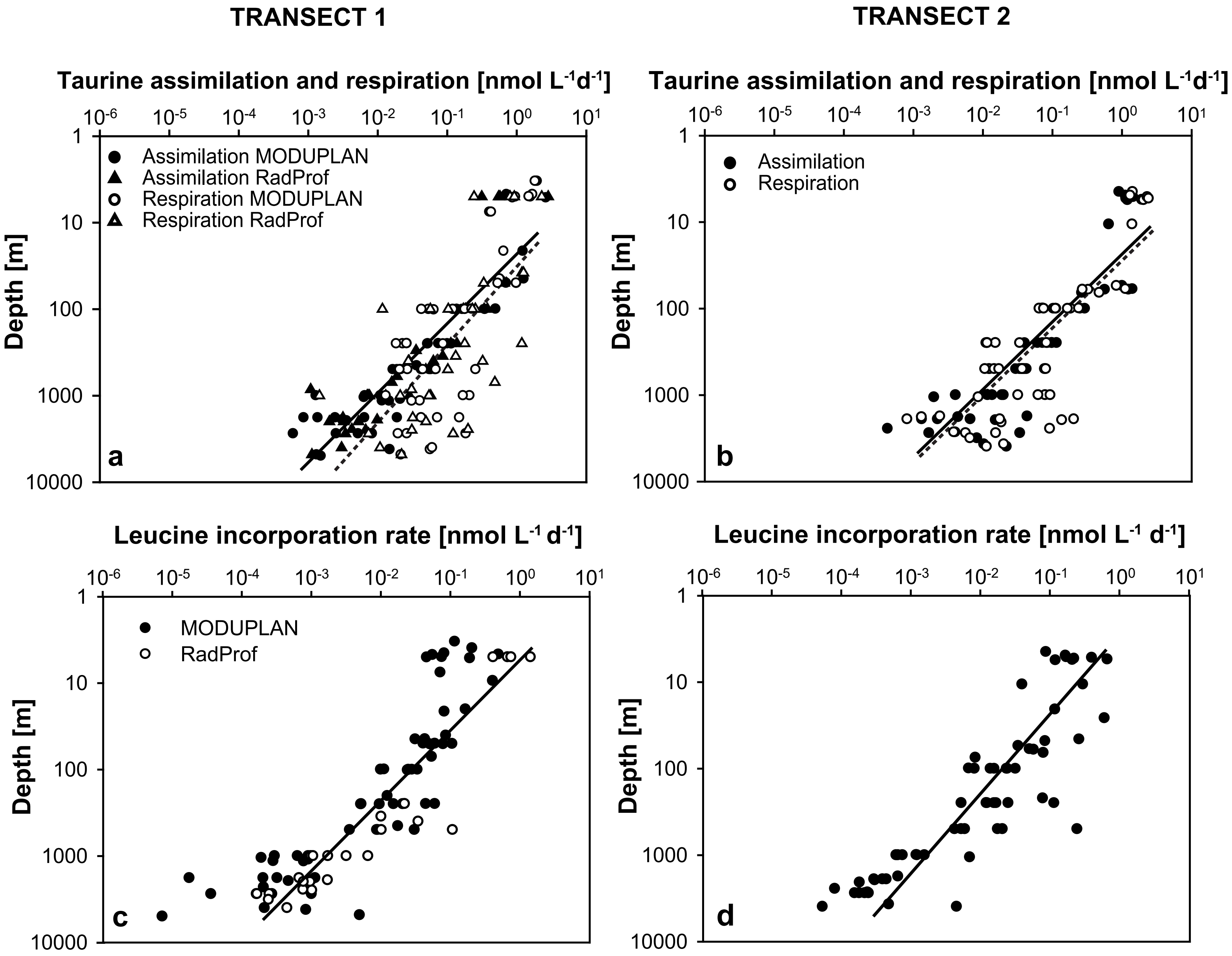

Fig. S8. Log – log plots of taurine assimilation and respiration rates (average of duplicates) throughout the water column of (a) Transect 1 and (b) Transect 2; leucine incorporation rates (average of duplicates) throughout the water column of (c) Transect 1 and (d) Transect 2 at the stations occupied during the MODUPLAN and the RadProf Cruise in the North Atlantic (Fig. 1). The full and dotted lines in a and b represent the trend-lines of taurine assimilation and respiration rates of the entire data sets with depth, respectively. The full lines in c and d represent the trend-lines of leucine incorporation rates with depth. The slopes and the coefficients of determination (R^2) for taurine assimilation and respiration and leucine incorporation rates are indicated in Table S5.

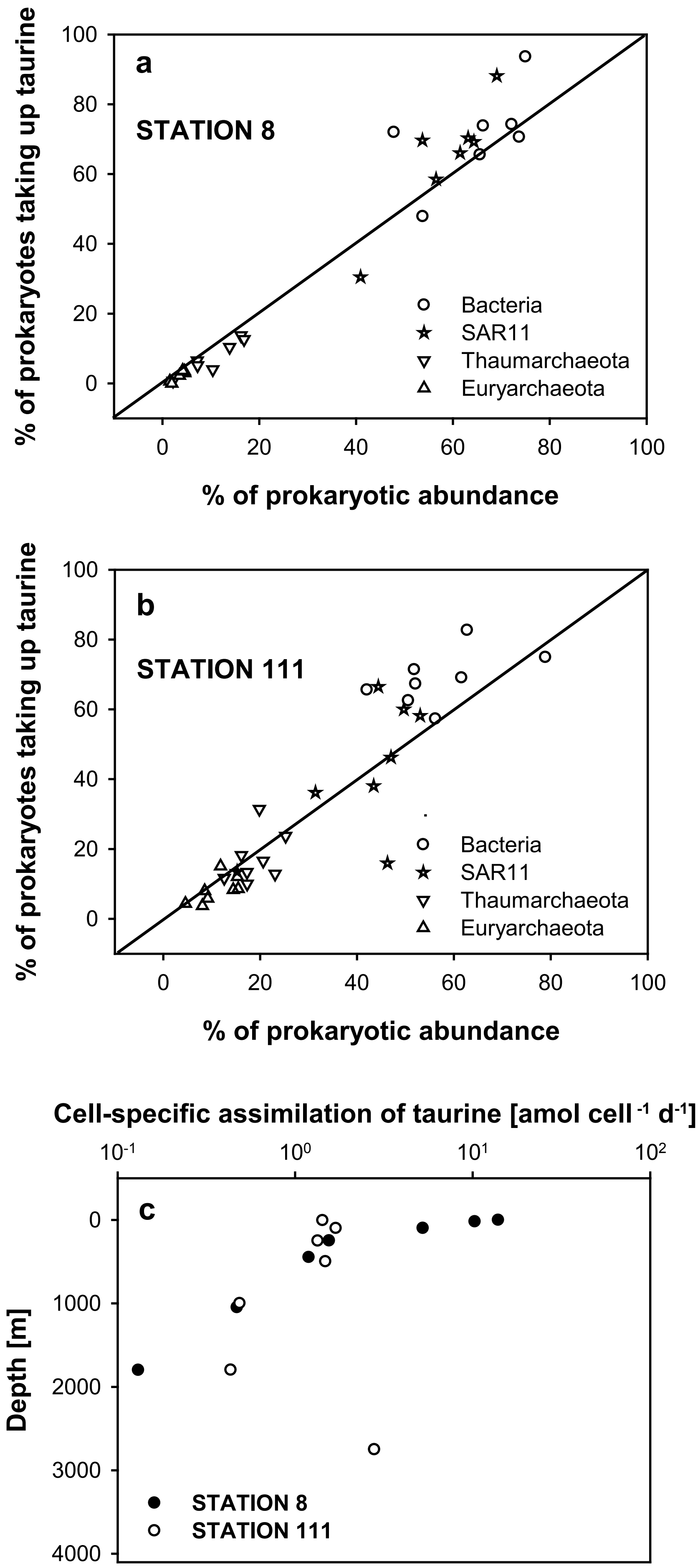

Fig. S9. The contribution of prokaryotes taking up taurine of the respective prokaryotic taxa *versus* their contribution to the abundance of prokaryotes (based on DAPI-counts): (a) Station 8, (b) Station 111. Cell-specific assimilation of taurine *versus* depth at Stations 8 and 111 (c)

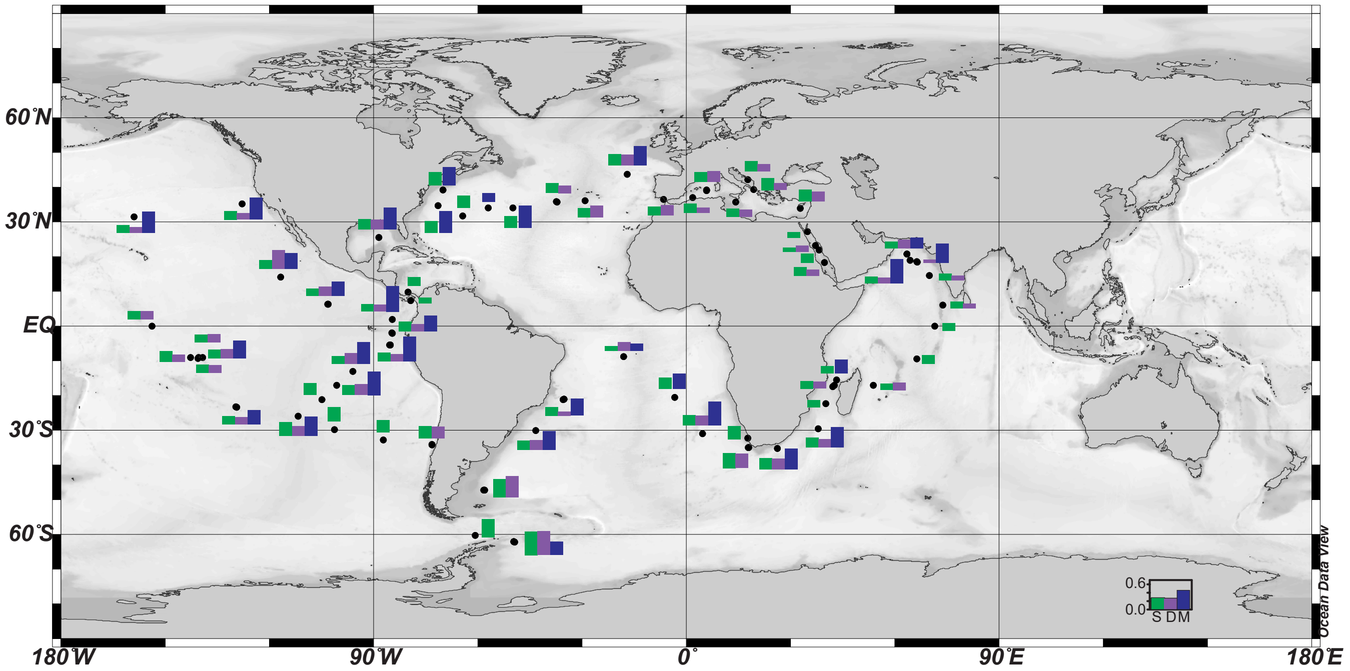

Fig. S10. Ratio between the abundance of taurine transporters (tauA + tauB + tauC) and the housekeeping gene of recombinases (recA and radA) in the Tara Oceans dataset [8]. Sampling locations are marked by full circles, bar charts depict the relative abundances for different depth layers according to the scale indicated in the graph in the bottom right corner. S, subsurface, D, deep chlorophyll maximum (DCM) (Table S3), M, mesopelagic

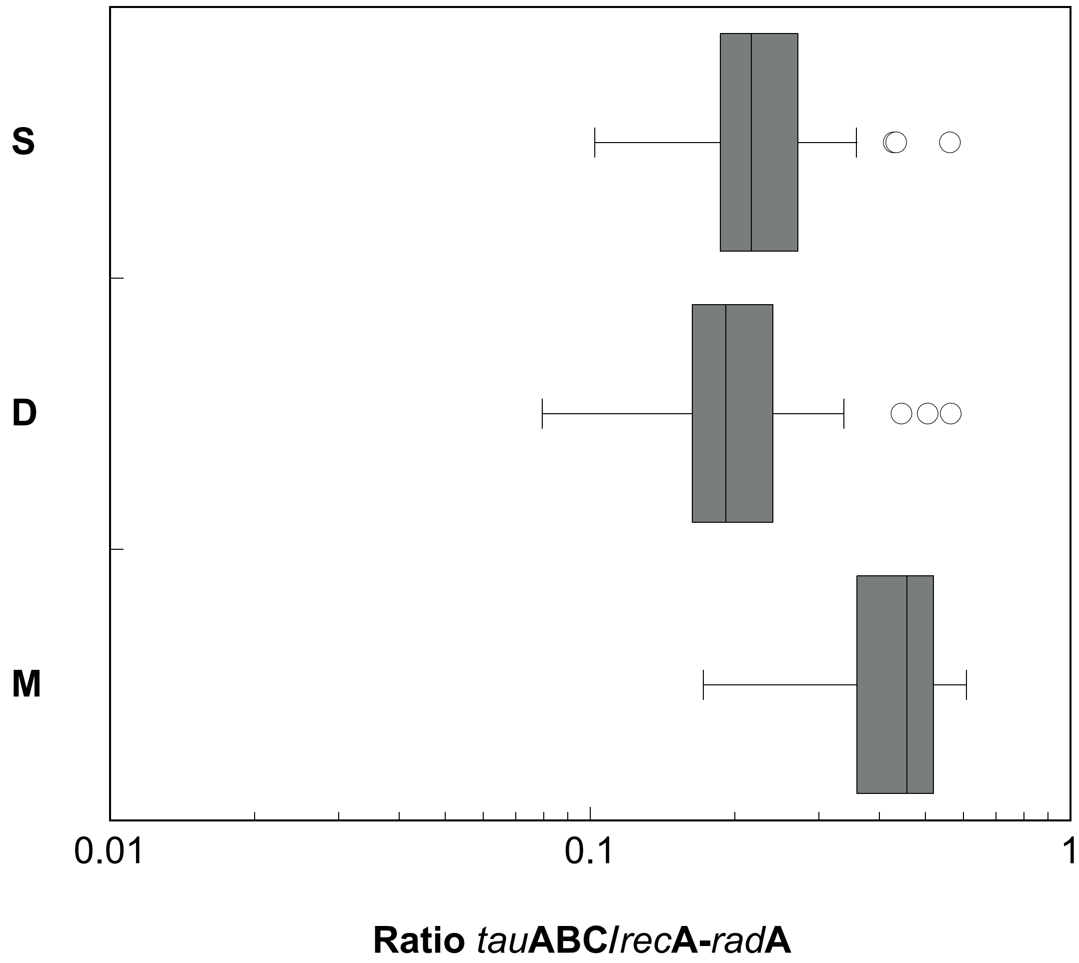

Fig. S11. Boxplot depicting the ratio between the sum of taurine transporters and *rec*A-*rad*A gene abundances in different depth layers of the Tara Oceans dataset [8]. The vertical line in the box represents the median, the lower box border represents the 25th percentile and the upper box border the 75th percentile, the whiskers represent the minimum and maximum values within the data set that fall within an acceptable range, and circles indicate outliers. The four samples identified as mixed layer in the dataset (Table S3) have been included in the DCM data. Abbreviations: S, subsurface water; D, deep chlorophyll maximum; M, mesopelagic.

1. Amann RI, Ludwig W, Schleifer KH (1995) Phylogenetic identification and in situ detection of individual microbial cells without cultivation. Microbiol Rev 59:143–69

2. Amann RI, Binder BJ, Olson RJ, et al (1990) Combination of 16S rRNA-targeted oligonucleotide probes with flow cytometry for analyzing mixed microbial populations. Appl Environ Microbiol 56:1919–25

3. Daims H, Brühl A, Amann R, et al (1999) The domain-specific probe EUB338 is insufficient for the detection of all Bacteria: development and evaluation of a more comprehensive probe set. Syst Appl Microbiol 22:434–44 . doi: 10.1016/S0723-2020(99)80053-8

4. Morris RM, Rappé MS, Connon SA, et al (2002) SAR11 clade dominates ocean surface bacterioplankton communities. Nature 420:806–810 . doi: 10.1038/nature01240

5. Teira E, Reinthaler T, Pernthaler A, et al (2004) Combining catalyzed reporter deposition-fluorescence in situ hybridization and microautoradiography to detect substrate utilization by bacteria and archaea in the deep ocean. Appl Environ Microbiol 70:4411–4414 . doi: 10.1128/AEM.70.7.4411-4414.2004

6. Woebken D, Fuchs BM, Kuypers MMM, Amann R (2007) Potential interactions of particle-associated anammox bacteria with bacterial and archaeal partners in the Namibian upwelling system. Appl Environ Microbiol 73:4648–4657 . doi: 10.1128/AEM.02774-06

7. Clifford EL, Hansell DA, Varela MM, et al (2017) Crustacean zooplankton release copious amounts of dissolved organic matter as taurine in the ocean. Limnol Oceanogr 62:2745–2758 . doi: 10.1002/lno.10603

8. Sunagawa S, Coelho LP, Chaffron S, et al (2015) Structure and function of the global ocean microbiome. Science 348:1261359 . doi: 10.1126/science.1261359
